# Galectin-3 inhibitor GB0139 protects against acute lung injury by inhibiting neutrophil recruitment and activation

**DOI:** 10.3389/fphar.2022.949264

**Published:** 2022-08-08

**Authors:** Duncan C. Humphries, Ross Mills, Cecilia Boz, Brian J. McHugh, Nikhil Hirani, Adriano G. Rossi, Anders Pedersen, Hans T. Schambye, Robert J. Slack, Hakon Leffler, Ulf J. Nilsson, Wei Wang, Tariq Sethi, Alison C. Mackinnon

**Affiliations:** ^1^ Centre for Inflammation Research, University of Edinburgh, Edinburgh, United Kingdom; ^2^ Galecto Inc. Nine Edinburgh BioQuarter, Edinburgh, United Kingdom; ^3^ Galecto Inc, Copenhagen, Denmark; ^4^ Galecto Inc, Stevenage, United Kingdom; ^5^ Department of Laboratory Medicine, Lund University, Lund, Sweden; ^6^ Department of Chemistry, Lund University, Lund, Sweden; ^7^ Department of Asthma, Allergy and Respiratory Science, King’s College London, Guy’s Hospital, London, United Kingdom

**Keywords:** galectin-3, acute lung injury, neutrophiils, cytokine, LPS

## Abstract

**Rationale:** Galectin-3 (Gal-3) drives fibrosis during chronic lung injury, however, its role in acute lung injury (ALI) remains unknown. Effective pharmacological therapies available for ALI are limited; identifying novel concepts in treatment is essential. GB0139 is a Gal-3 inhibitor currently under clinical investigation for the treatment of idiopathic pulmonary fibrosis. We investigate the role of Gal-3 in ALI and evaluate whether its inhibition with GB0139 offers a protective role. The effect of GB0139 on ALI was explored *in vivo* and *in vitro.*

**Methods:** The pharmacokinetic profile of intra-tracheal (*i.t.*) GB0139 was investigated in C57BL/6 mice to support the daily dosing regimen. GB0139 (1–30 µg) was then assessed following acute *i.t.* lipopolysaccharide (LPS) and bleomycin administration. Histology, broncho-alveolar lavage fluid (BALf) analysis, and flow cytometric analysis of lung digests and BALf were performed. The impact of GB0139 on cell activation and apoptosis was determined *in vitro* using neutrophils and THP-1, A549 and Jurkat E6 cell lines*.*

**Results:** GB0139 decreased inflammation severity via a reduction in neutrophil and macrophage recruitment and neutrophil activation. GB0139 reduced LPS-mediated increases in interleukin (IL)-6, tumor necrosis factor alpha (TNFα) and macrophage inflammatory protein-1-alpha. *In vitro*, GB0139 inhibited Gal-3-induced neutrophil activation, monocyte IL-8 secretion, T cell apoptosis and the upregulation of pro-inflammatory genes encoding for IL-8, TNFα, IL-6 in alveolar epithelial cells in response to mechanical stretch.

**Conclusion:** These data indicate that Gal-3 adopts a pro-inflammatory role following the early stages of lung injury and supports the development of GB0139, as a potential treatment approach in ALI.

## Introduction

Galectin-3 (Gal-3) is a pro-fibrotic, mammalian ß-galactoside binding lectin, which is highly upregulated in the injured lung (MacKinnon et al., 2012). Gal-3 is elevated in the plasma and broncho-alveolar lavage fluid (BALf) of patients with idiopathic pulmonary fibrosis (IPF) (Nishi et al., 2007; MacKinnon et al., 2012), and is further upregulated in the plasma of patients undergoing an acute exacerbation of IPF (AE-IPF) (MacKinnon et al., 2012). Furthermore, in a cohort of 2025 patients from the Framingham Heart study, elevated plasma Gal-3 was associated with restrictive lung disease, decreased lung volumes and altered gas exchange (Ho et al., 2016), suggesting a potential role for Gal-3 in the early stages of pulmonary fibrosis.

Preclinical data supports the role of Gal-3 as an important regulator of lung fibrosis; global deletion of Gal-3 in mice was shown to reduce bleomycin-induced fibrosis compared to wild-type mice (MacKinnon et al., 2012). *In vitro* findings also suggest a role for Gal-3 in fibrogenesis as Gal-3 stimulates migration and collagen synthesis in fibroblasts (Nishi et al., 2007), and promotes alternative, pro-fibrotic, macrophage activation (MacKinnon et al., 2008). Gal-3 is also a key regulator in the induction of epithelial to mesenchymal transition (EMT) in lung epithelial cells (MacKinnon et al., 2012), and has a role in neutrophil activation and neutrophil apoptosis (Yamaoka et al., 1995; Kuwabara and Liu, 1996; Almkvist and Karlsson, 2002; Farnworth et al., 2008; Sundqvist et al., 2018). In mouse models of inflammation, elevated levels of Gal-3 in exudates correlates with increased neutrophil recruitment to the inflammatory site (Sano et al., 2000); such persistent neutrophil activation and delay of apoptosis could result in an overall exacerbation of tissue injury and failure of resolution.

In lung epithelial cells, Gal-3 activates ERK, AKT and JAK/STAT1 signaling pathways, leading to the release of pro-inflammatory cytokines during influenza and *Streptococcus pneumoniae* co-infection (Nita-Lazar et al., 2015), and enhances the pathogenic effects of H5N1 avian influenza virus by promoting host inflammatory responses via an interaction with NLRP3 inflammasome in macrophages (Chen et al., 2018). Mice deficient in Gal-3 develop less severe inflammation and interleukin (IL)-1β production than wild-type mice (Chen et al., 2018). In dendritic cells (DC) Gal-3 serves as a pattern-recognition receptor, regulating proinflammatory cytokine production and downregulation of Gal-3 in DCs inhibits expression of IL-6, IL-1β, and IL-23 and subsequent Th17 and Th2 development (Chen et al., 2015).

Currently, no targeted therapies exist for ALI and so treatment is limited to best supportive care. Based on available data, the combined effects of Gal-3 on macrophages, lung epithelial cells and neutrophil function, suggest that the inhibition of Gal-3 may serve as a potential strategy for the treatment of ALI. Recently we showed that conditional myeloid deletion of Gal-3 led to a significant reduction in Gal-3 expression in alveolar macrophages and neutrophils which decreased pulmonary inflammation and neutrophil recruitment into the interstitium (Humphries et al., 2021). GB0139 (formerly TD139), is a novel, inhalable, small molecule Gal-3 inhibitor, which reduces bleomycin-induced fibrosis in mice (MacKinnon et al., 2012; Delaine et al., 2016). In a Phase I/IIa study, GB0139 had a manageable safety profile and demonstrated good target engagement with alveolar macrophages in patients with IPF (Hirani et al., 2021). GB0139 is currently undergoing Phase IIb clinical evaluation for the treatment of IPF (NCT03832946). Here, we investigate the impact of Gal-3 and GB0139, on ALI models in mice, and on neutrophil and epithelial cell activation *in vitro.*

## Methods and materials

### *In-vivo* studies

*Animals:* 8-week-old male C57BL/6 mice were purchased from Harlan (Harlan Ltd, United Kingdom) and given 1 week to acclimatize prior to experimentation. Mice were maintained in 12-h light/12-h dark cycles with free access to food and water. All experimental animal procedures were approved by the University of Edinburgh and were performed in accordance with Home Office guidelines [Animal (Scientific Procedures) Act 1986].

#### GB0139 pharmacokinetics

For full details on GB0139 lung and plasma pharmacokinetics following intra-tracheal (*i.t*.) delivery see online supplement.

#### Induction of ALI and administration of GB0139

To induce ALI, mice received 10 μg lipopolysaccharide ([LPS] serotype 0127:B8, L4516, Sigma-Aldrich, Missouri, United States) from *E. coli*, or 33 μg bleomycin (BI3543, Apollo Scientific, United Kingdom), respectively, via *i.t.* administration. LPS/bleomycin ± GB0139 (in 50 μL 0.9% NaCl), was inserted into the needle via a pipette, and delivered into the lungs with a 2 × 100 μL bolus of air (using a 1 ml syringe). GB0139 was subsequently administered every 24 h until sacrifice.

#### Bronchoalveolar lavage

BALf was collected as previously described (Dhaliwal et al., 2012).

##### Histology and immunohistochemistry preparation

For full details of histology and immunohistochemistry preparation, see online supplement. Total inflammation score and fibrosis score were assessed according to protocols (Murao et al., 2003; Hübner et al., 2008). Quantitative analysis of histological and immunohistochemical samples was performed blinded to the investigator.

#### Total protein

Total protein within BALf was performed using a Pierce BCA Total Protein Assay Kit (23227; ThermoFisher Scientific, Waltham, MA, United States) as per the manufacturer’s instructions.

#### Flow cytometric analysis of lung digests

Tissue digests and flow cytometry methods were performed according to published methodology (Humphries et al., 2018). See online supplement for further details.

#### Cytokine analysis

The mouse magnetic luminex assay (LXSAMSM, R&D Systems, Minneapolis, MN, United States) was used according to the manufacturer’s instructions.

#### Enzyme Linked immunosorbent assay (ELISA)

ELISA kit for the measurement of IL-8 or Gal-3 in BALf samples (DuoSet; R&D Systems) was used according to the manufacturer’s instructions.

#### Gal-3 synthesis

Gal-3 was synthesized in house. Recombinant human full length Gal-3 was produced in *E. Coli* BL21 Star (DE3) cells and purified by affinity chromatography on lactosyl-sepharose columns, as previously described (Salomonsson et al., 2010). To remove endotoxin contamination, 1% Triton X114 (X114, Sigma-Aldrich) was added to Gal-3 solution for 30 min (min) at 4°C prior to 10 min incubation at 37°C. After centrifugation at 5000 *g* for 5 min, the aqueous solution was removed. This process was repeated 3 times. GB0139 (Bis (3-deoxy-3-(3-fluorophenyl-1*H*-1,2,3-triazol-1-yl)-β-d-galactopyranosyl) sulfane) was provided by Galecto Inc. The purity of GB0139 was >99% as determined by analytical high-performance liquid chromatography.

#### Cell culture

Cell lines (THP-1, A549 and Jurkat E6 cells) were purchased from the European Collection of Authenticated Cell Cultures and were cultured at 37°C in 5% CO_2_ (95% air) in Dulbecco’s Modified Eagle’s Medium (A549) or Roswell Park Memorial Institute medium (THP1, Jurkat E6) supplemented with 10% fetal calf serum, 1% l-glutamine, and 1% penicillin/streptomycin.

#### Monocyte IL-8 secretion

THP-1 monocyte-like cells (ATCC, Middlesex, United Kingdom) were activated with 100 nM phorbol 12-myristate 13-acetace ([PMA] P8139, Sigma-Aldrich) overnight and allowed to adhere. The following day cells were washed x three with phosphate buffered saline (PBS) and treated with Gal-3 ± 10 µM GB0139 for 24 h. Levels of IL-8 within media were quantified using IL-8 ELISA (DY208, R&D Systems).

#### Isolation of human neutrophils/monocytes

Peripheral human neutrophils and mononuclear cells were isolated from whole blood using Percoll gradients (Dorward et al., 2017). To isolate monocytes, the pan monocyte isolation kit (130-096-537, Miltenyi Biotec, Germany) was used as per the manufacturer’s instructions.

#### Macrophage RNA analysis

For full details of macrophage polarization and RNA analysis, see online supplement.

#### Luminol ROS assay

Human peripheral neutrophils were primed with 10 ng/ml tumor necrosis factor alpha ([TNFα] 210-TA, R&D Systems) for 30 min at 37°C prior to addition of GB0139 for a further 10 min. To quantify reactive oxygen species (ROS) release, neutrophils were mixed with Horse Radish Peroxidase (P8375, Sigma-Aldrich)/Luminol (A8511, Sigma-Aldrich) and incubated with 30 μg/ml Gal-3, with luminescence measured using a Synergy plate reader (BioTek, Winooski, VT, United States).

#### Neutrophil/Jurkat apoptosis

Neutrophils or Jurkat cells were cultured for 20 h in the presence of 10 μg/ml or 20 μg/ml Gal-3, respectively, ± GB0139. Rates of apoptosis were determined using Annexin V (11828681001, Roche, Switzerland)/PI (P4170, Sigma-Aldrich) staining. Samples were assessed using the FACSCalibur flow cytometer (BD Biosciences, United Kingdom) and analyzed using FlowJo software (Tree Start Inc., OR, United States).

#### Stretch-induced gene changes in human lung epithelial cells in vitro

Human lung epithelial A549 cells were plated in 6-well collagen Bioflex culture plates (Flexcell International Corporation, Germany) ± 10 μg/ml Gal-3 ± 10 µM GB0139. Cyclic mechanical stretch was applied using a Flexcell FX-4000T Tension Plus system (Flexcell International Corporation, Germany) set to deliver 15% elongation at 1 Hz for 4 h. After the stretch procedure, RNA was isolated from the cells and analyzed by quantitative polymerase chain reaction array. For full details of the gene set see online supplement.

#### Statistics

Data are represented as mean ± the standard error of the mean (SEM). Statistical comparisons were made using two-tailed Students t-test or one-way/two-way analysis of variance (ANOVA) with Bonferroni post-test for multiple comparisons. A *p* value <0.05 was considered statistically significant (* = *p* < 0.05, ** = *p* < 0.01, *** = *p* < 0.001, **** = *p* < 0.0001). All graphs and statistics were performed using the statistical package GraphPad Prism five for Windows (GraphPad Software, CA, United States).

## Results

### GB0139 pharmacokinetic data support *i.t.* daily dosing

**FIGURE 1:**
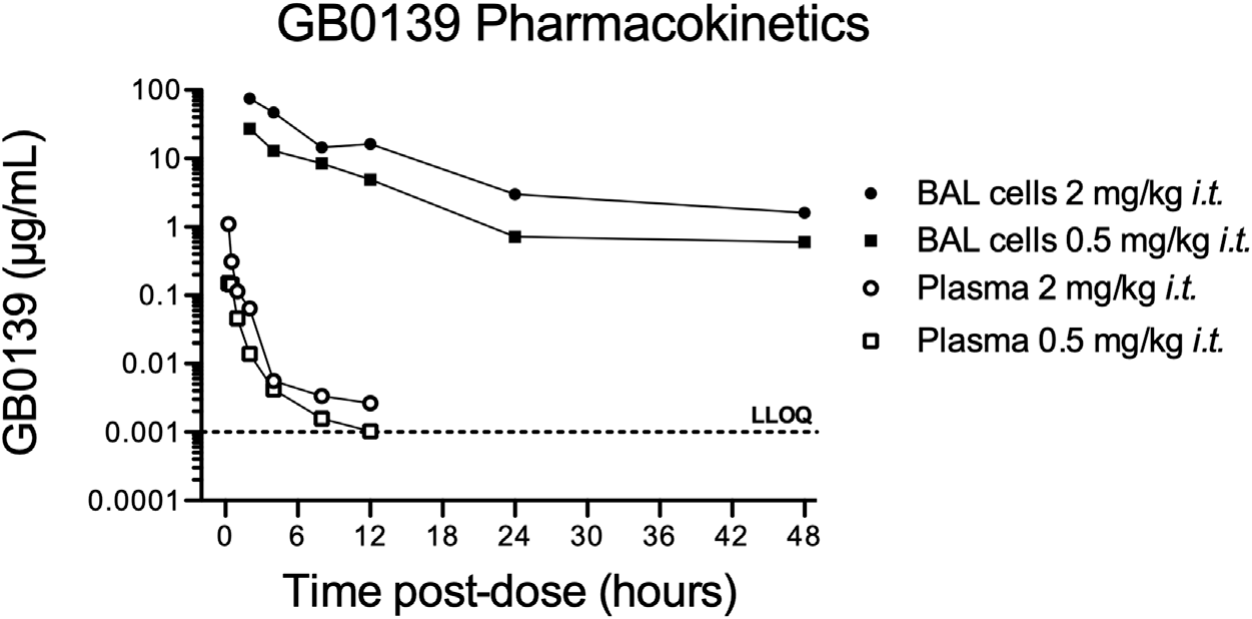
Pharmacokinetic profile of GB0139 in female C57BL/6 mice following *i.t.* administration. BAL cell and total blood concentrations of GB0139 were determined by LC-MS/MS overtime following single *i.t.* Administrations of 0.5 mg/kg and 2 mg/kg. Data shown are the mean values of three animals in each time point. BAL = broncho-alveolar lavage; *i.t.* = intra-tracheal; LC-MS/MS = liquid chromatography-tandem mass spectrometry; LLOQ = lowest level of quantification (1 ng/ml).

The pharmacokinetic profile of GB0139 was assessed in naïve mice. GB0139 was retained at high concentrations in the lung for up to 48 h following *i.t.* dosing of 0.5 mg/kg and 2 mg/kg (Figure 1). This supported daily dosing of 0.3–1 mg/kg (9–30 μg per mouse) to be taken forward into ALI models.

### GB0139 reduces inflammatory cell recruitment following LPS-induced ALI

**FIGURE 2:**
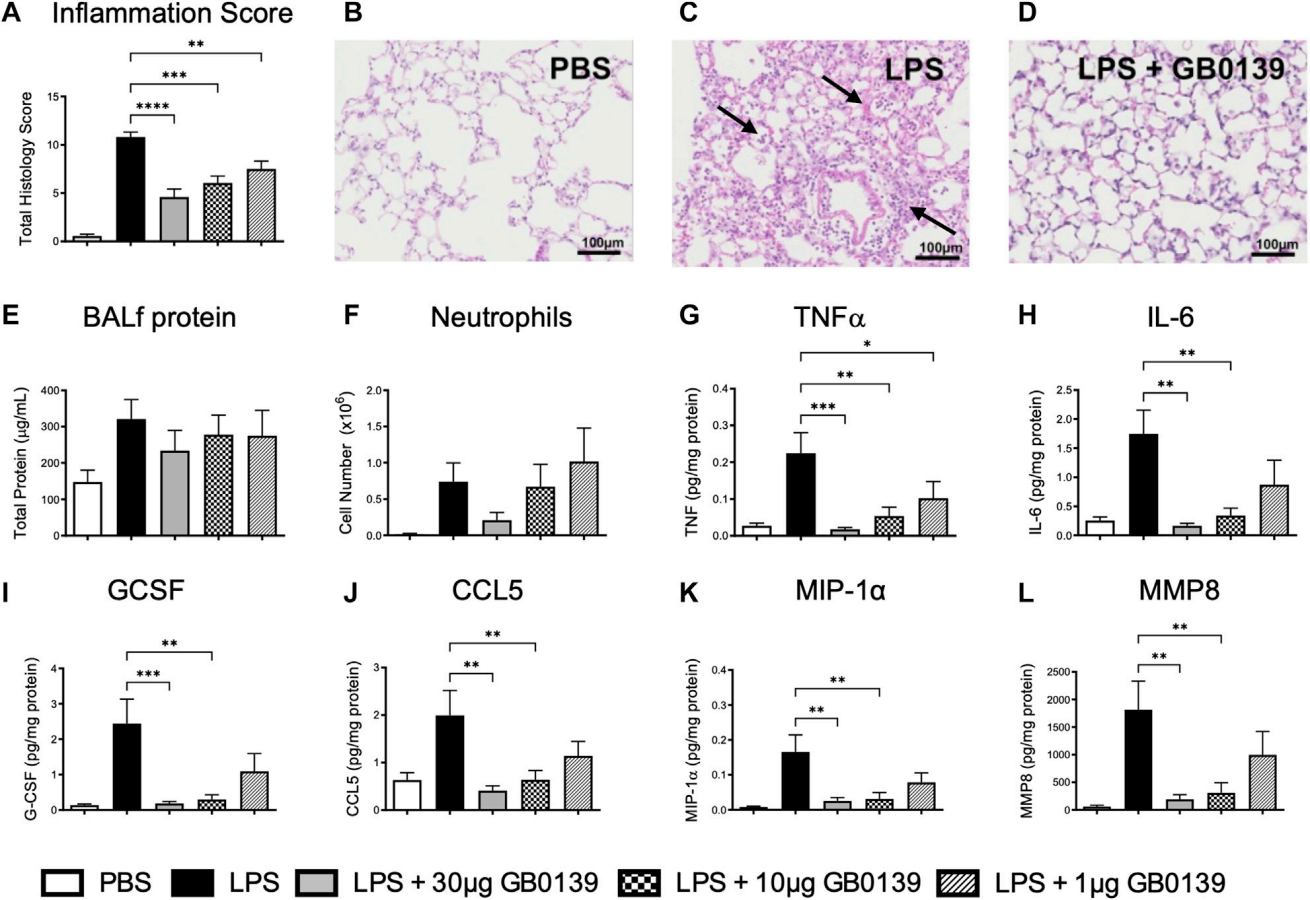
Histological and BALf analysis following LPS-induced lung inflammation. 10 µg LPS was administered alongside 1, 10 or 30 μg GB0139 *i.t.* and tissue retrieved 24 h later. Cytokine levels were normalised against total BALf protein. **(A–D)** Histology inflammation score and representative images of H and E stained lung tissue sections. Arrows indicate areas of pulmonary inflammation (alveolar membrane thickening, capillary congestion and alveolar neutrophil infiltration). Inflammation score was based on the presence of alveolar membrane thickening, capillary congestion, intra-alveolar haemorrhage, interstitial neutrophil infiltration and alveolar neutrophil infiltration. **(E)** BALf total protein. **(F)** BALf neutrophil numbers. **(G–J)** BALf pro-inflammatory cytokine profiles. **(K–L)** BALf pro-fibrotic cytokine profiles. Data represented as mean ± SEM. Analysed via 1-way ANOVA (n = 6, *p < 0.05, **p < 0.01, ***p < 0.001, ****p < 0.0001). Images taken at ×200 magnification. ANOVA = analysis of variance; BALf = broncho-alveolar lavage fluid; CCL = C-C motif chemokine ligand; E = eosin; G-CSF = granulocyte-colony stimulating factor; H = hematoxylin; h = hours; IL = interleukin; *i.t.* = intra-tracheal; LPS = lipopolysaccharide; MIP-1α = macrophage inflammatory protein-1-alpha; MMP = matrix metallopeptidase; PBS = phosphate bufferd saline; SEM = standard error of the mean; TNF = tumor necrosis factor.

LPS administration resulted in significant pulmonary inflammation at 24 h, as seen by alveolar membrane thickening, capillary congestion, intra-alveolar hemorrhage and interstitial and alveolar neutrophil infiltration. Histology inflammation score was significantly reduced in a dose-dependent manner with GB0139 when compared with the LPS treatment only group (Figure 2A−D). No significant differences in vascular permeability were observed, however a trend of increased BALf total protein was seen following LPS, which was partially reduced following GB0139 (Figure 2E). Although absent following PBS, neutrophils were detected within the alveolar space following LPS administration and were reduced with 30 μg GB0139 (Figure 2F).

LPS administration upregulated several pro-inflammatory and pro-fibrotic cytokines within BALf, however administration of 10–30 μg GB0139 significantly reduced TNFα, IL-6, granulocyte-colony stimulating factor, C-C motif chemokine ligand (CCL) 5, macrophage inflammatory protein-1-alpha (MIP-1α), and matrix metallopeptidase 8 (MMP8) in a dose-dependent manner (Figure 2G–L, Supplementary Figure S1A, B).

**FIGURE 3:**
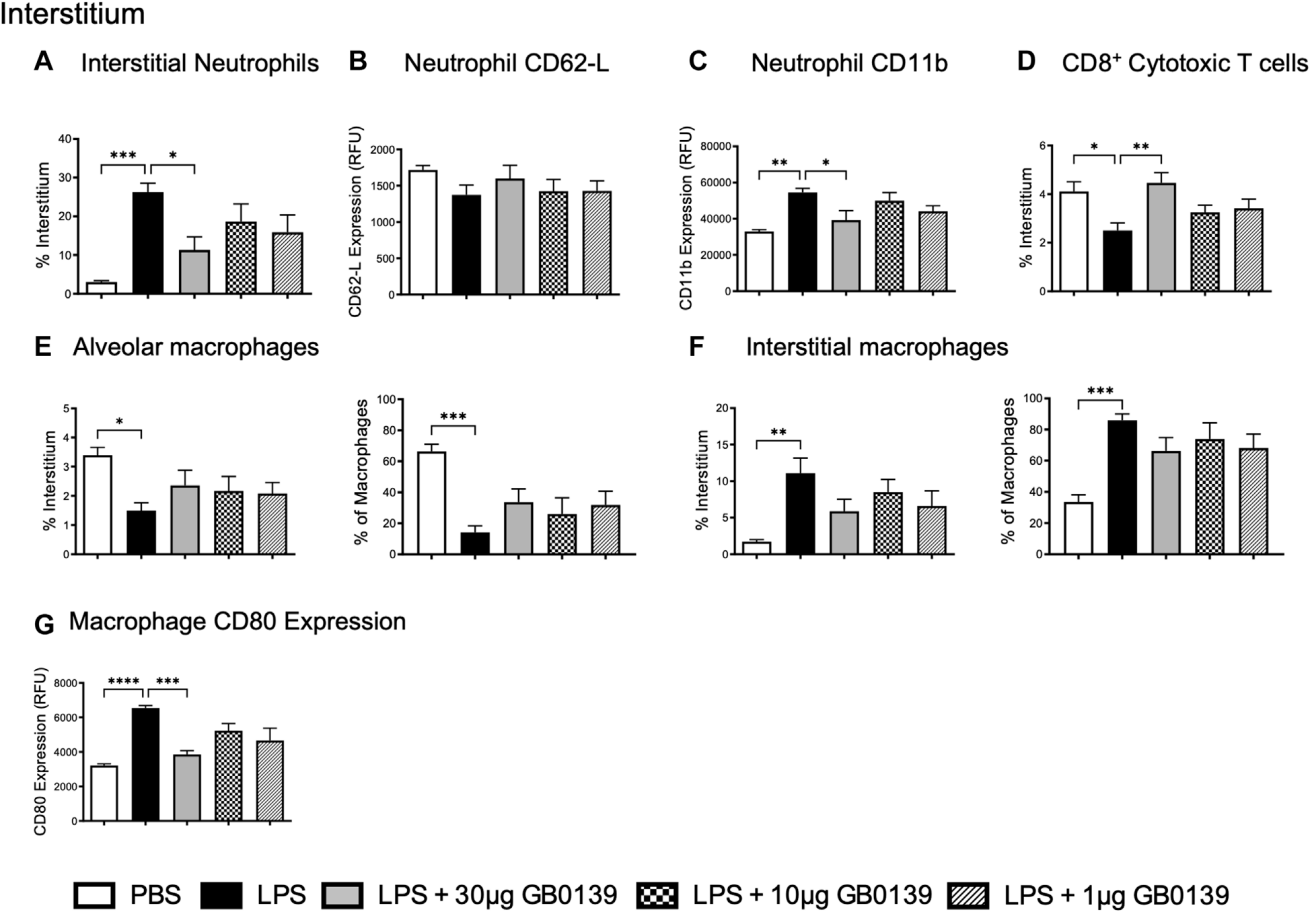
Flow cytometric analysis of whole lung digests following LPS-induced lung inflammation. 10 µg LPS was administered alongside 1, 10 or 30 μg GB0139 *i.t.* and tissue retrieved 24 h later. **(A–C)** Interstitial neutrophil numbers and activation parameters (CD62L and CD11b). **(D)** Interstitial CD8^+^ cytotoxic T-cell accumulation. **(E–F)** Alveolar and interstitial macrophage accumulation, and **(G)** CD80 expression. Data represented as mean ± SEM. Analysed via 1-way ANOVA (n = 6, *p < 0.05, **p < 0.01, ***p < 0.001, ****p < 0.0001). ANOVA = analysis of variance; h = hours; *i.t.* = intra-tracheal; LPS = lipopolysaccharide; RFU = relative fluorescence units; SEM = standard error of the mean.

The reduction of pulmonary inflammation seen with 30 μg GB0139 was associated with a significant decrease in interstitial neutrophil recruitment (identified as CD11b^+^, LY-6G^+^) and activation, as seen by a significant reduction in CD11b expression (Figures 3A–C) – for gating strategies see Supplementary Figures S2−4. A significant reduction in interstitial cytotoxic T cells (CD3^+^, CD8^+^) was also seen with LPS, which was reversed with 30 μg GB0139 (Figure 3D).

To identify alveolar and interstitial macrophage populations, a flow cytometric staining protocol was used (Misharin et al., 2013). LPS-induced ALI significantly reduced alveolar macrophage numbers whilst increasing inflammatory interstitial macrophage recruitment (measured as both % of total interstitial cells and proportion of total macrophages) (Figures 3E,F). Both effects were partially inhibited with 30 μg GB0139, although the data were not statistically significant. CD80, a marker of inflammatory M1 macrophages, was seen to increase following LPS treatment and was significantly reduced with 30 μg GB0139 (Figure 3G).

Similar results were also seen at the 48 h timepoint, with GB0139 reducing histology inflammation score, pulmonary neutrophil number and activation (Supplementary Figure S5A−C). A significant increase in the proportion of alveolar macrophages alongside a significant reduction in interstitial macrophage recruitment was observed with GB0139 compared with the LPS only group (Supplementary Figure S5D, E). Analysis of the prototypical markers for M1 (CD80) and M2 (CD206) macrophages showed that Gal-3 inhibition was also found to non-significantly reduce CD80 expression on both alveolar and interstitial macrophages, whilst significantly increasing CD206 expression on interstitial macrophages. This suggests Gal-3 inhibition may have an M2-mediated protective role following LPS-induced ALI.

**FIGURE 4:**
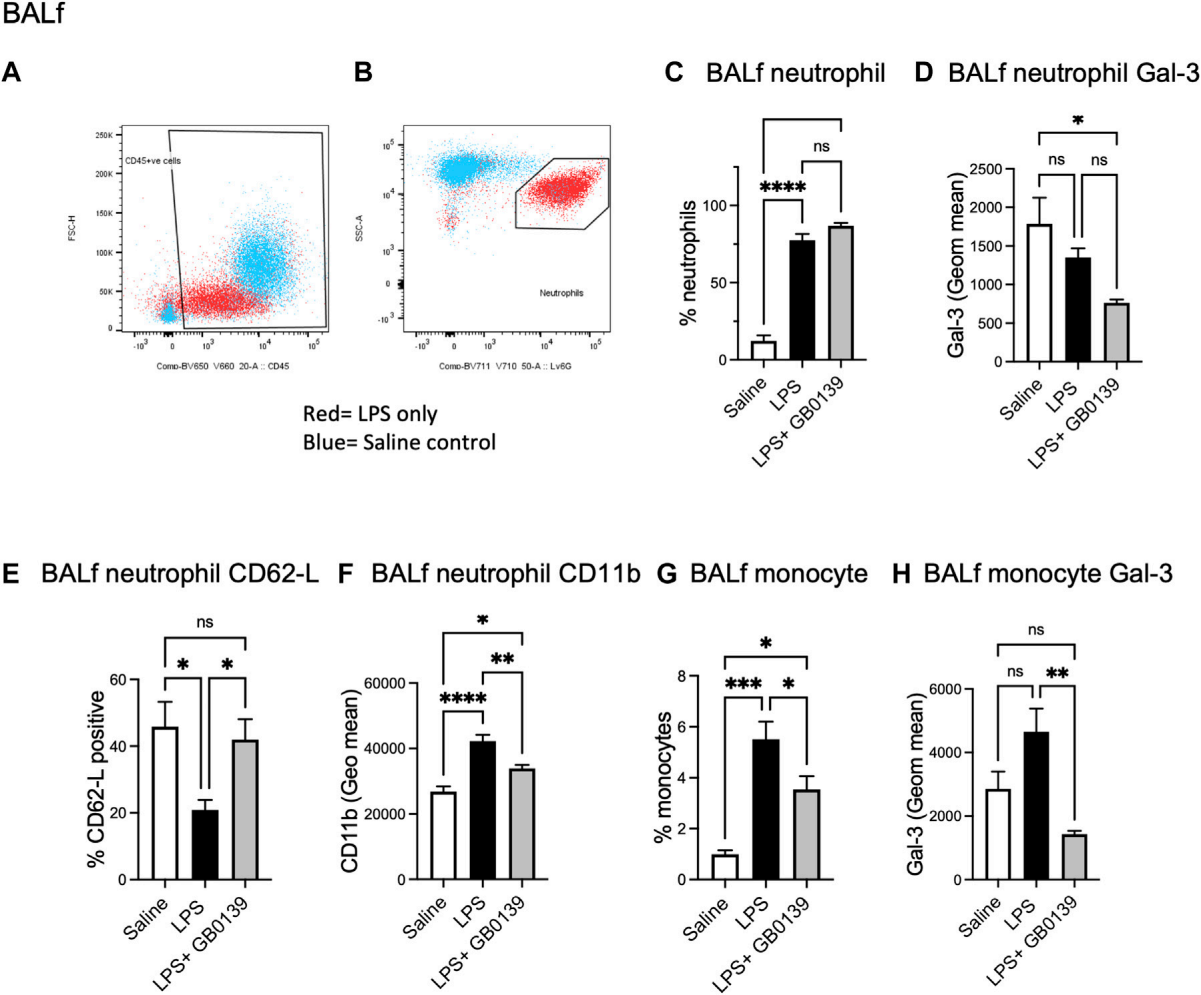
Flow cytometric analysis of BALf following LPS-induced lung inflammation. 10 µg LPS was administered alongside 30 μg GB0139 *i.t.* and tissue retrieved 24 h later. **(A–B)** Gating strategy to identify CD45^+^ cells (left panel) and LY-6G^+^ neutrophils (right panel) in BALf. **(C)** BALf neutrophil numbers, **(D)** Gal-3 expression and **(E–F)** activation parameters (CD62L and CD11b). **(G–H)** BALf monocytes and Gal-3 expression. Data represented as mean ± SEM. Analysed via 1-way ANOVA (n = 6, *p < 0.05, **p < 0.01, ***p < 0.001, ****p < 0.0001). ANOVA = analysis of variance; BALf = broncho-alveolar lavage fluid; Gal-3 = galectin-3; h = hours; *i.t.* = intra-tracheal; LPS = lipopolysaccharide; SEM = standard error of the mean.

Flow cytometric analysis of BALf (Figures 4A–D) revealed GB0139 treatment did not affect the overall numbers of neutrophils but did reduce neutrophil activation as measured by a increase in neutrophil CD62L and decrease in CD11b expression when compared with LPS-treated mice (Figures 4E,F). Cell surface Gal-3 expression (geometric mean fluorescence intensity measured via flow cytometry) was also decreased by GB0139 and there was a reduction in both recruitment and Gal-3 expression on CD11b^+^, LY-6G^−^, inflammatory monocytes in BALf (Figures 4G,H).

### Bleomycin-induced acute lung injury

**FIGURE 5:**
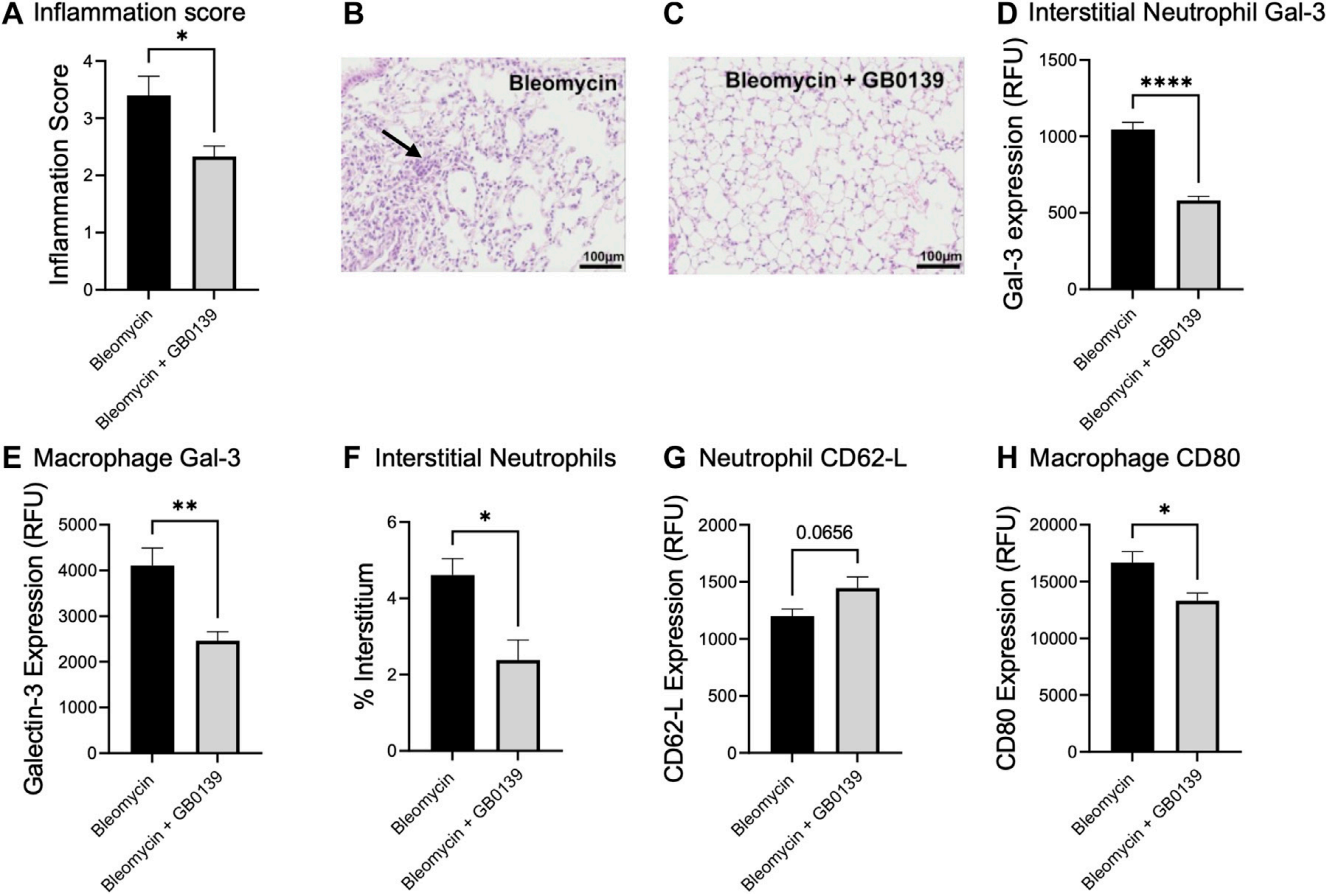
Effect of GB0139 on bleomycin-induced lung inflammation. 33 μg bleomycin was administered *i.t.* with further administration of 30 μg GB0139 every 24 h and lung tissue retrieved at day 3. **(A–C)** Histology inflammation score and representative H and E sections following administration of bleomycin with/without GB0139. Arrow indicates areas of pulmonary inflammation (alveolar membrane thickening, capillary congestion and alveolar neutrophil infiltration). **(D)** Gal-3 expression on interstitial neutrophils. **(E)** Gal-3 expression on macrophages. **(F)** Interstitial neutrophil accumulation. **(G)** Expression of CD62L on neutrophils. **(H)** Expression of the M1 marker, CD80 on macrophages. Data represented as mean ± SEM. Analysed via Student’s t-test (n = 5, *p < 0.05, **p < 0.01, ***p < 0.001, ****p < 0.0001). Images taken at ×200 magnification. E = eosin; H = haematoxylin; h = hours; *i.t.* = intra-tracheal; RFU = relative fluorescence units; SEM = standard error of the mean.

We have previously shown that global genetic deletion of Gal-3 and therapeutic administration of GB0139 reduces chronic inflammation and fibrosis induced by bleomycin (MacKinnon et al., 2012; Delaine et al., 2016). We examined the effect of GB0139 on the acute inflammatory phase following bleomycin injury in mice treated daily with 30 µg GB0139. Following bleomycin-induced injury, significantly lower inflammation scores were observed at day 3 in the GB0139-treated group versus the bleomycin only group (Figures 5A–C). Flow cytometric analysis of lung digests showed that GB0139 significantly reduced Gal-3 expression on interstitial neutrophils and macrophages and reduced interstitial neutrophil accumulation following bleomycin (Figures 5D–F). GB0139 reduced neutrophil activation as determined by an increase in CD62L expression (Figure 5G) and reduced inflammatory M1 macrophage polarization (decrease in CD80 expression; Figure 5H).

### GB0139 has anti-inflammatory properties *in vitro*

**FIGURE 6:**
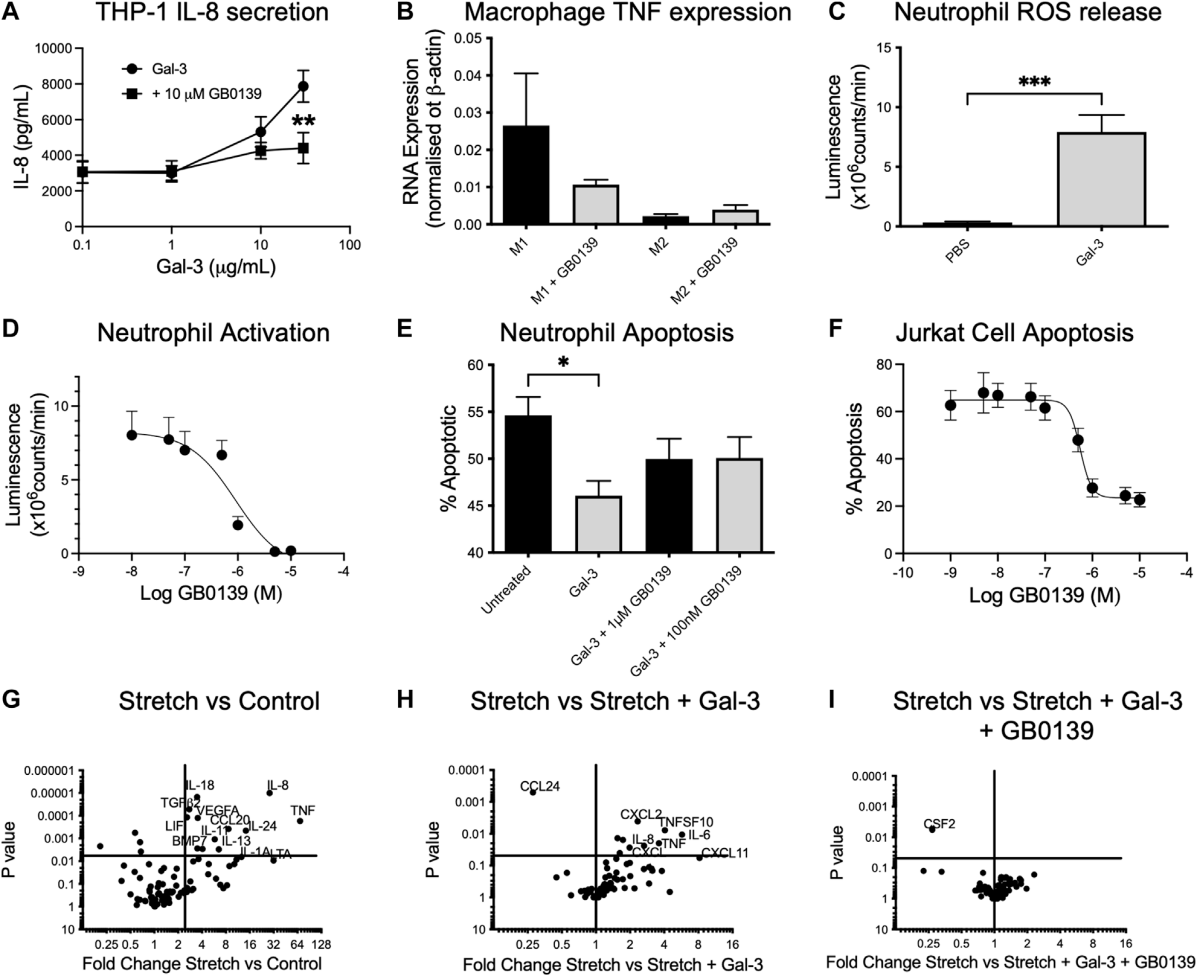
Effects of GB0139 *in vitro*. **(A)** THP-1 monocyte IL-8 secretion. THP-1 cells were differentiated with 10 nM PMA and treated with human recombinant Gal-3 ± 10 µM GB0139 for 24 h. **(B)** Human monocyte-derived macrophages were cultured in the presence of GM-CSF (M1) or M-CSF (M2) for 6 days and then further activated with IFN-*γ*/LPS (M1) or IL-4 (M2) ± 10 µM GB0139 for 48 h. **(C)** Neutrophil ROS production. Neutrophils were primed with TNFα (10 ng/ml), prior to stimulation with 30 μg/ml Gal-3. **(D)** GB0139 inhibition of Gal-3-induced neutrophil activation. **(E)** Neutrophil apoptosis. Neutrophils were cultured for 20 h in the presence of 10 μg/ml Gal-3 ± GB0139. **(F)** Jurkat cell apoptosis. Jurkat cells were cultured for 20 h in the presence of 20 μg/ml Gal-3 ± GB0139. Data represented as mean ± SEM. Analyzed via 2-way ANOVA (Figure 6A), 1-way ANOVA (Figures 6B,E), students t-test (Figure 6C) (n = 4–9, *p < 0.05, **p < 0.01, ***p < 0.001). The effects of stretch-induced gene expression in human lung epithelial cells following GB0139 treatment are shown in Figure 6G−I. Human epithelial A549 cells underwent a period of cyclic mechanical stretch ±10 μg/ml Gal-3 and/or 10 µM GB0139 prior to gene analysis. **(G)** Gene expression in human lung epithelial cells following mechanical stretch. **(H)** Effect of Gal-3 on stretch-induced changes in gene expression. **(I)** Effect of GB0139 on Gal-3-induced gene expression following stretch. Data (gene expression fold-change associated *p* value) represented as a dot plot. ANOVA = analysis of variance; Gal-3 = galectin-3; GM-CSF = granulocyte macrophage colony stimulating factor; h = hours; IFN-*γ* = interferon gamma; IL = interleukin; LPS = lipopolysaccharide; PMA = phorbol 12-myristate 13-acetate; ROS = reactive oxygen species; SEM = standard error of the mean; TNFα = tumor necrosis factor alpha.

We sought to define the mechanism of action of GB0139 on cell types known to drive ALI. *In vitro* addition of 10–30 μg/ml Gal-3 to human THP-1 monocyte-like cells led to a significant increase in IL-8 secretion, which was inhibited with 10 µM GB0139 (Figure 6A). Human monocyte-derived macrophages upregulated TNFα gene expression when polarized towards an M1 phenotype by culturing in the presence of LPS and interferon gamma (IFNγ), which was reduced with 10 µM GB0139 (Figure 6B). Human neutrophils primed with TNFα increased ROS production in response to 30 μg/ml Gal-3 (Figure 6C). This was significantly inhibited with GB0139 (IC_50_ 0.8 µM; Figure 6D). Gal-3 also significantly delayed rates of neutrophil apoptosis that was partially inhibited with GB0139 (Figure 6E). The opposite was seen in Jurkat cells (an immortalized human T-cell line). Gal-3 (20 μg/ml) significantly increased apoptosis from 11% to 65% (Figure 6F). This was inhibited with GB0139 (IC_50_ 0.54 μM).

Epithelial cells play a key role in inflammation and repair in the diseased lung. To assess the effects of epithelial damage *in vitro*, human lung epithelial (A549) cells were exposed to a period of cyclic mechanical stretch. Stretch induced a significant upregulation of several genes encoding for pro-inflammatory cytokines, in particular IL-8, IL-6 and TNFα, and chemokines such as CCL20 and regulatory molecules (vascular endothelial growth factor, transforming growth factor beta-2) (Figure 6G). The addition of 10 μg/ml Gal-3 produced a further upregulation of IL-6, IL-8 and TNFα, and chemokines C-X-C motif chemokine ligand 1 (CXCL1) and CXCL2 (Figure 6H). Co-incubation with 10 µM GB0139 abolished these Gal-3-mediated changes and further reduced colony-stimulating factor-2 (CSF2) expression (Figure 6I). Gal-3 inhibition therefore reduces inflammation by inhibiting neutrophil activation, accelerating neutrophil apoptosis and inhibiting pro-inflammatory M1 macrophage activation, whilst reducing pro-inflammatory cytokine release from injured epithelial cells.

## Discussion

ALI is a condition with limited treatment options and so identifying novel therapeutic targets is essential. The accumulation and activation of neutrophils is considered key to the progression of ALI into the life-threatening acute respiratory distress syndrome (ARDS) (Abraham, 2003). This study examined the effect of the Gal-3 inhibitor, GB0139, on LPS- and bleomycin-induced ALI in mice and explored its mechanism of action *in vitro* in human inflammatory cell types known to trigger ALI. When evaluated *in vivo* and *in vitro*, GB0139 was found to decrease inflammation severity whilst accelerating neutrophil apoptosis to promote resolution. Gal-3 may serve as a potential therapeutic target for ALI and should be explored further.

GB0139 reduced interstitial neutrophil accumulation following LPS-induced lung inflammation. GB0139 was found to reduce both interstitial and alveolar neutrophil recruitment at least at the highest dose of GB0139. This is in keeping with our previous observations that global Gal-3 deletion reduced neutrophil recruitment into both compartments, whereas myeloid specific deletion only impacted interstitial recruitment (Humphries et al., 2021). This would suggest that GB0139 can additionally inhibit Gal-3 in the alveolar space derived from other non-myeloid cells (Humphries et al., 2021). Gal-3 derived from stromal cells has also been shown to mediate neutrophil extravasation into the alveolar space following *Aspergillus fumigatus* infection (Snarr et al., 2020). We also show that GB0139 inhibits Gal-3-induced delay of neutrophil apoptosis, which may be one mechanism whereby GB0139 reduces LPS-induced inflammation. The expression of Gal-3 and the number of inflammatory monocytes recruited into the alveolar space was also inhibited by GB0139. The reduction in CSF2 expression in injured alveolar epithelial cells suggests a mechanism whereby GB0139 may inhibit monocyte and neutrophil recruitment into the lung.

GB0139 also demonstrates affinity for Gal-1 (MacKinnon et al., 2012; Peterson et al., 2018). It is therefore conceivable that GB0139 may also target Gal-1 in the lung, however, we have not shown significant upregulation of Gal-1 in the BALf or on the surface of BAL cells following LPS or bleomycin acute injury (data not shown). Our view is that the activated cells that are recruited into the lung in response to LPS or bleomycin injury have elevated Gal-3 (principally from monocytes). GB0139 inhibits recruitment and activation of these cells and as a result Gal-3 is itself reduced whereas the level of soluble Gal-1 in BALf is not elevated by injury or inhibited by GB0139. In addition, the effect we see is similar to that seen in the global Gal-3 deficient mouse (Humphries et al., 2021) so we conclude the effect of GB0139 is largely down to inhibition of Gal-3. Increased Gal-1 has however been associated with worse prognosis in other interstitial lung disease (ILD) (d’Alessandro et al., 2020) and COVID-19 induced inflammation (Markovic et al., 2022). We would therefore surmise that a co-inhibition of galectin-1 would have a beneficial effect although this requires further study.

We have shown that Gal-3 deletion and inhibition of Gal-3 with GB0139 inhibits IL-4-induced M2 macrophage activation (MacKinnon et al., 2008). However, in human monocytes, exogenously added Gal-3 induces production of superoxide (Liu et al., 1995) and acts as an autocrine ligand for toll-like receptor (TLR)4 and induces TLR4-mediated activation (Burguillos et al., 2015). We show that GB0139 reduced the predominance of the pro-inflammatory M1 phenotype, as seen via a reduction in CD80 expression on interstitial macrophages and reduced the recruitment of inflammatory monocytes into the alveolar space. Therefore, in response to M1 macrophage stimuli, Gal-3 may adopt a pro-inflammatory role. Previous studies have shown that Gal-3 binding to M1 and M2 macrophages has differential carbohydrate dependence (Lepur et al., 2012). Based on our findings, we propose that GB0139 inhibits M1 macrophage responses during acute injury whilst reducing the profibrotic M2 macrophage phenotype during chronic injury. Modulation of macrophage polarization has important implications during ALI such as in AE-IPF where cytokines produced by both M1-and M2-like macrophages are elevated (Schupp et al., 2015).

In addition, GB0139 maintained alveolar macrophage numbers, which have been shown to inhibit neutrophil recruitment following LPS-induced lung injury (Beck-Schimmer et al., 2005), preserved CD8^+^ T-cell populations and inhibited T-cell apoptosis *in vitro*. GB0139 may have a role in preserving CD8^+^ T-cell function and so promote the resolution of inflammation. Plasma levels of Gal-3 are significantly elevated in patients with COVID-19 infection (de Biasi et al., 2020) and it has been suggested that GB0139 may have utility in reducing viral-induced lung injury and preventing fibrosis following COVID-19 infection (George et al., 2020).

GB0139 inhibited several pro-inflammatory cytokines/chemokines, including IL-6, IL-8 and TNFα, which are considered typical biomarkers of ALI (Parsons et al., 2005). IL-8 predicts ARDS following major trauma (Donnelly et al., 1993; Folkesson et al., 1995) and is considered one of the most potent neutrophil chemo-attractants in inflammation (Hoffmann et al., 2002), and blocking IL-8 has been shown to protect rabbits from acid-aspiration-induced lung injury (Folkesson et al., 1995). Here, we show that GB0139 inhibits Gal-3-induced IL-8 secretion from monocytes. IL-6 is an important cytokine in ALI and stimulates profibrotic M2 macrophage activation during the fibrotic phase of bleomycin-injury (Ayaub et al., 2017). IL-6 may therefore be an important mediator of pro-fibrotic signaling in response to an acute injury. GB0139 decreased several pro-fibrotic mediators in the BALf including MMP8, tissue inhibitor matrix metalloproteinase 1 (TIMP-1) and MIP-1α, which are indicative of an early fibrotic signature (Marshall et al., 2000; Kolb et al., 2001). This suggests that GB0139 may inhibit an early fibrotic response to an acute injury.

Consistent with our findings, clinical data from a Phase I/IIa clinical trial in patients with IPF demonstrated that inhaled GB0139 impacts on several key fibrotic mediators in the lung. As well as having an acceptable safety profile, GB0139 is able to reach the alveolar compartment to reduce alveolar macrophage Gal-3 expression and reduce biomarkers associated with IPF progression. GB0139 has a manageable safety profile and is associated with a favorable cytokine profile in patients with SARS-CoV-2 infection (Gaughan et al., 2021).

In conclusion, our data show that when Gal-3 levels are high (such as following ALI), GB0139 decreases inflammation and promotes resolution by reducing inflammatory cell recruitment and pro-inflammatory cytokine release whilst accelerating neutrophil apoptosis. These data indicate a potential opportunity to exploit Gal-3 as a therapeutic target in ALI and support the progression of GB0139 into the Phase IIb GALACTIC-1 study (NCT03832946) in patients with IPF.

## Data Availability

The raw data supporting the conclusions of this article will be made available by the authors, without undue reservation.
