# Supplementary figures and images for: Galectin-3 inhibitor GB0139 protects against acute lung injury by inhibiting neutrophil recruitment and activation
Source: Front Pharmacol. 2022 Aug 8;13:949264. doi: 10.3389/fphar.2022.949264 (PMC9393216; doi:10.3389/fphar.2022.949264)

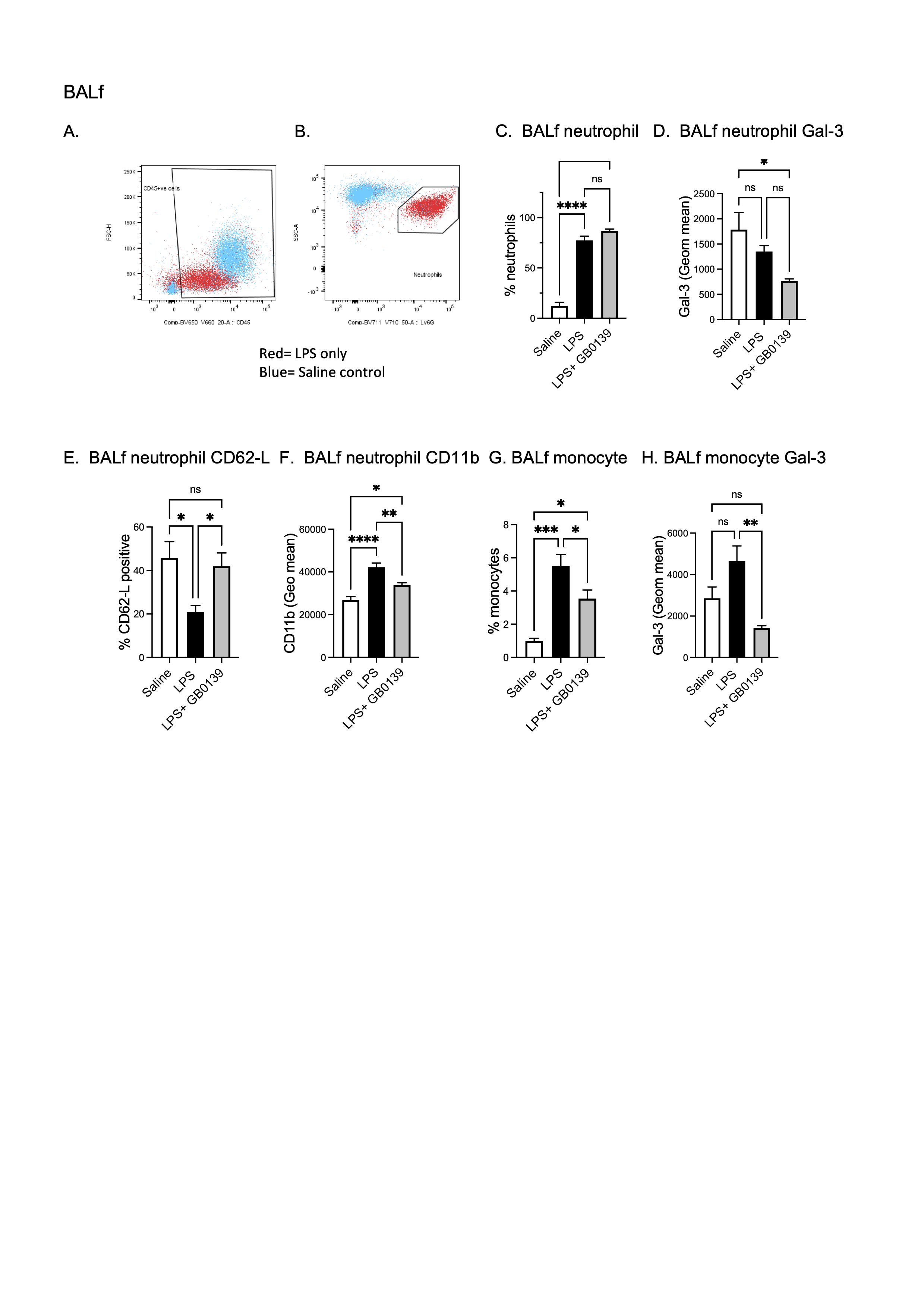

Supplement: Supplementary file 1 [file Presentation4.zip › figure 4.tiff]

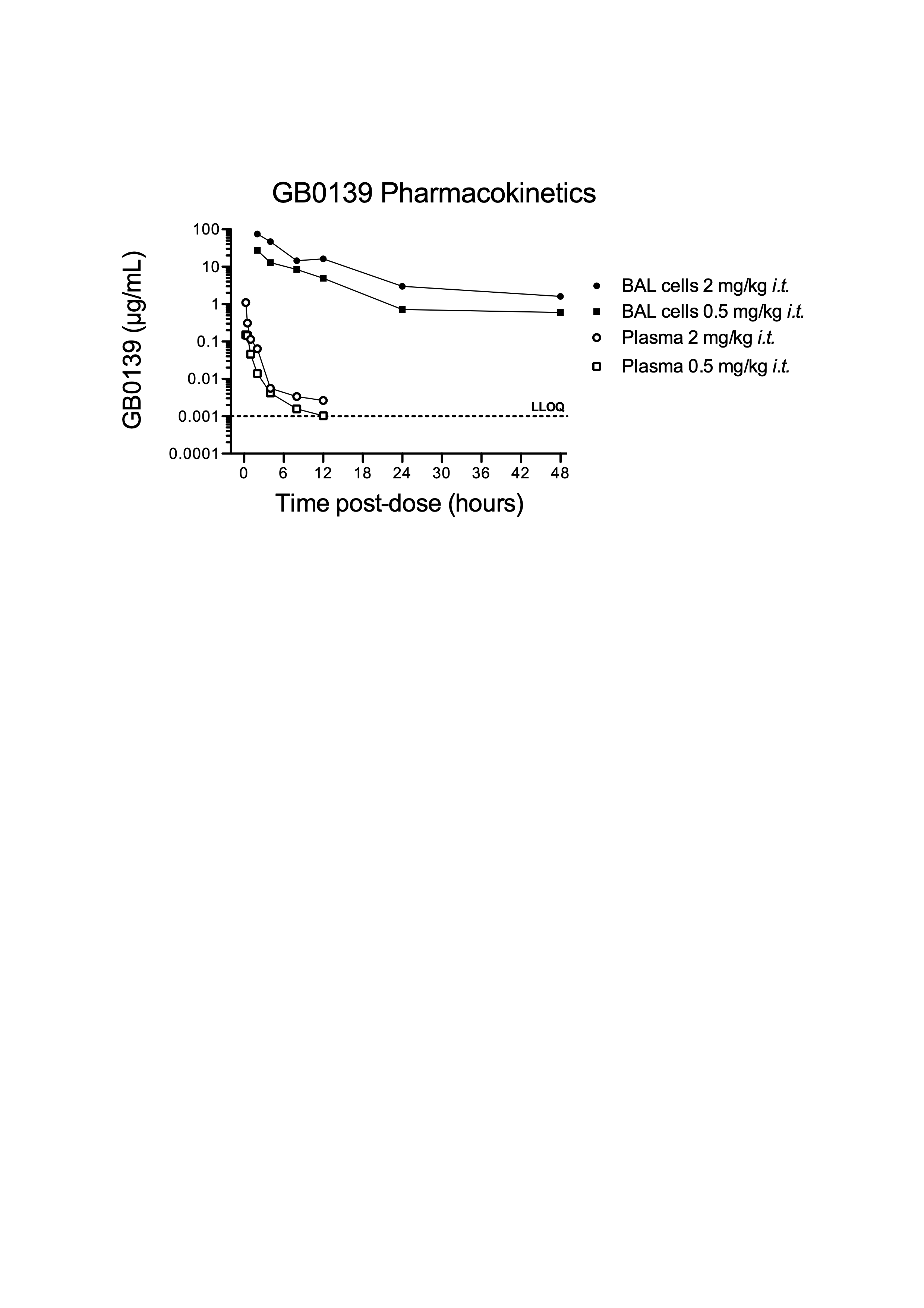

Supplement: Supplementary file 2 [file Presentation1.zip › figure 1.tiff]

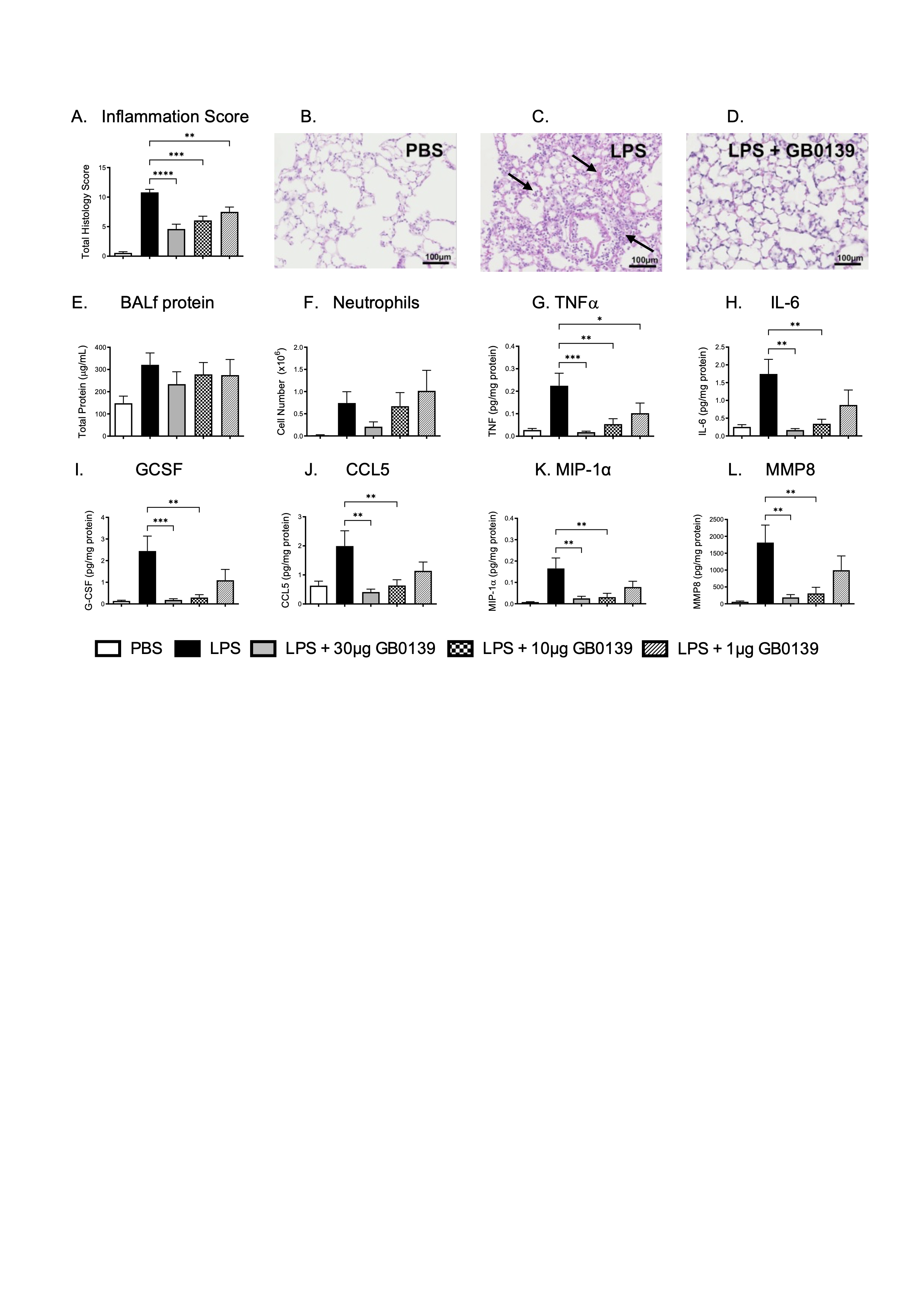

Supplement: Supplementary file 3 [file Presentation2.zip › figure 2.tiff]

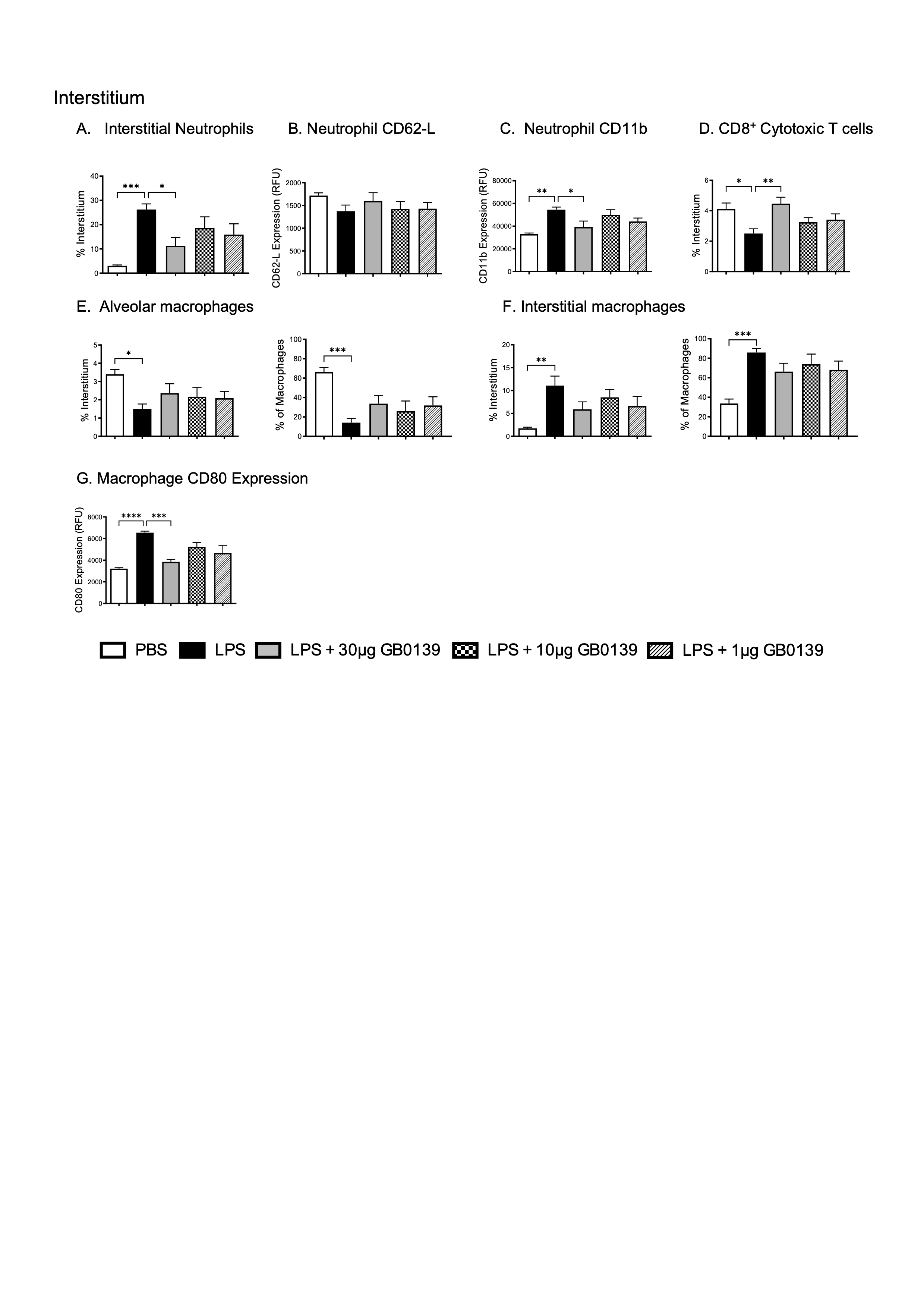

Supplement: Supplementary file 4 [file Presentation3.zip › figure 3.tiff]

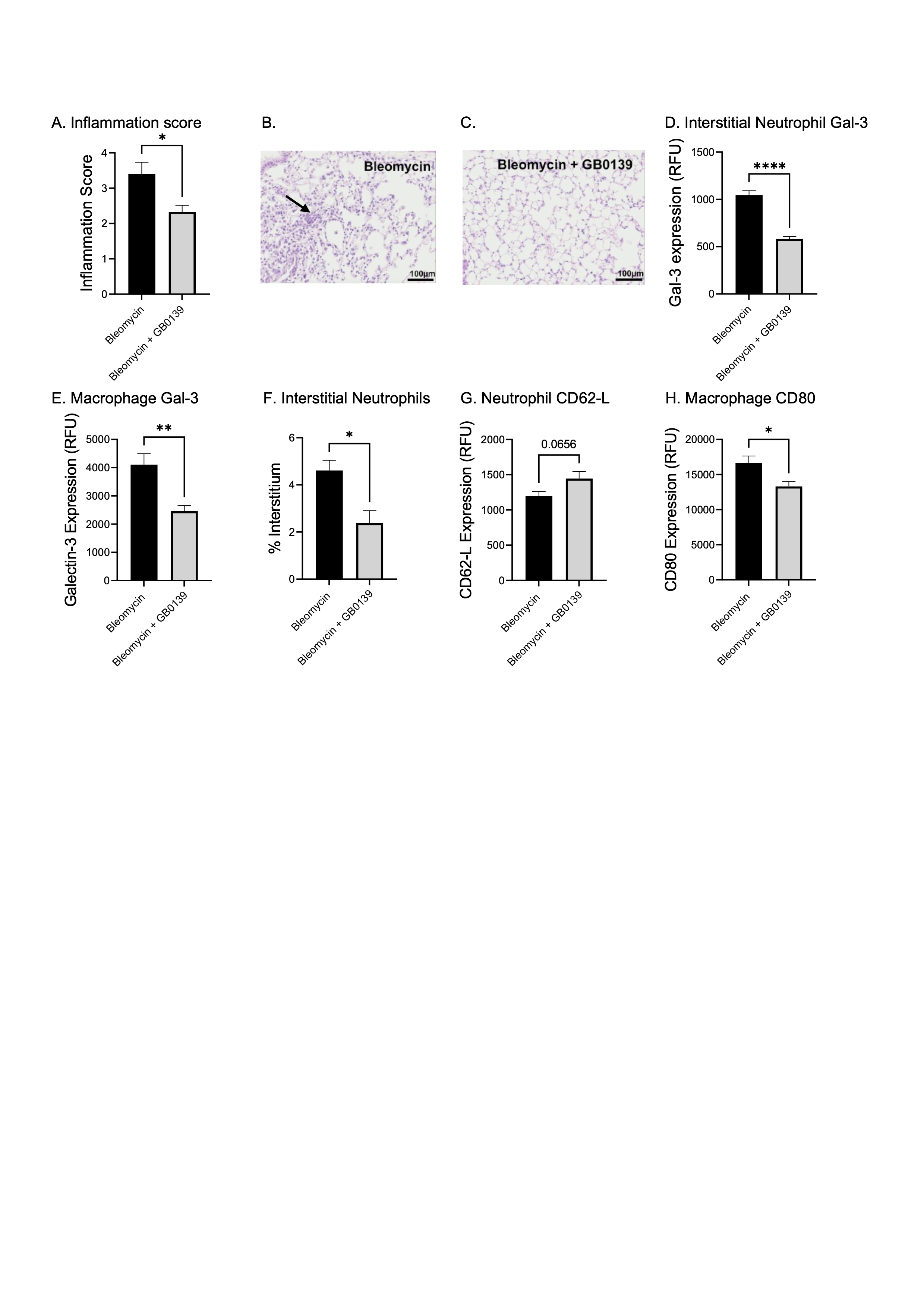

Supplement: Supplementary file 6 [file Presentation5.zip › Figure 5.tiff]

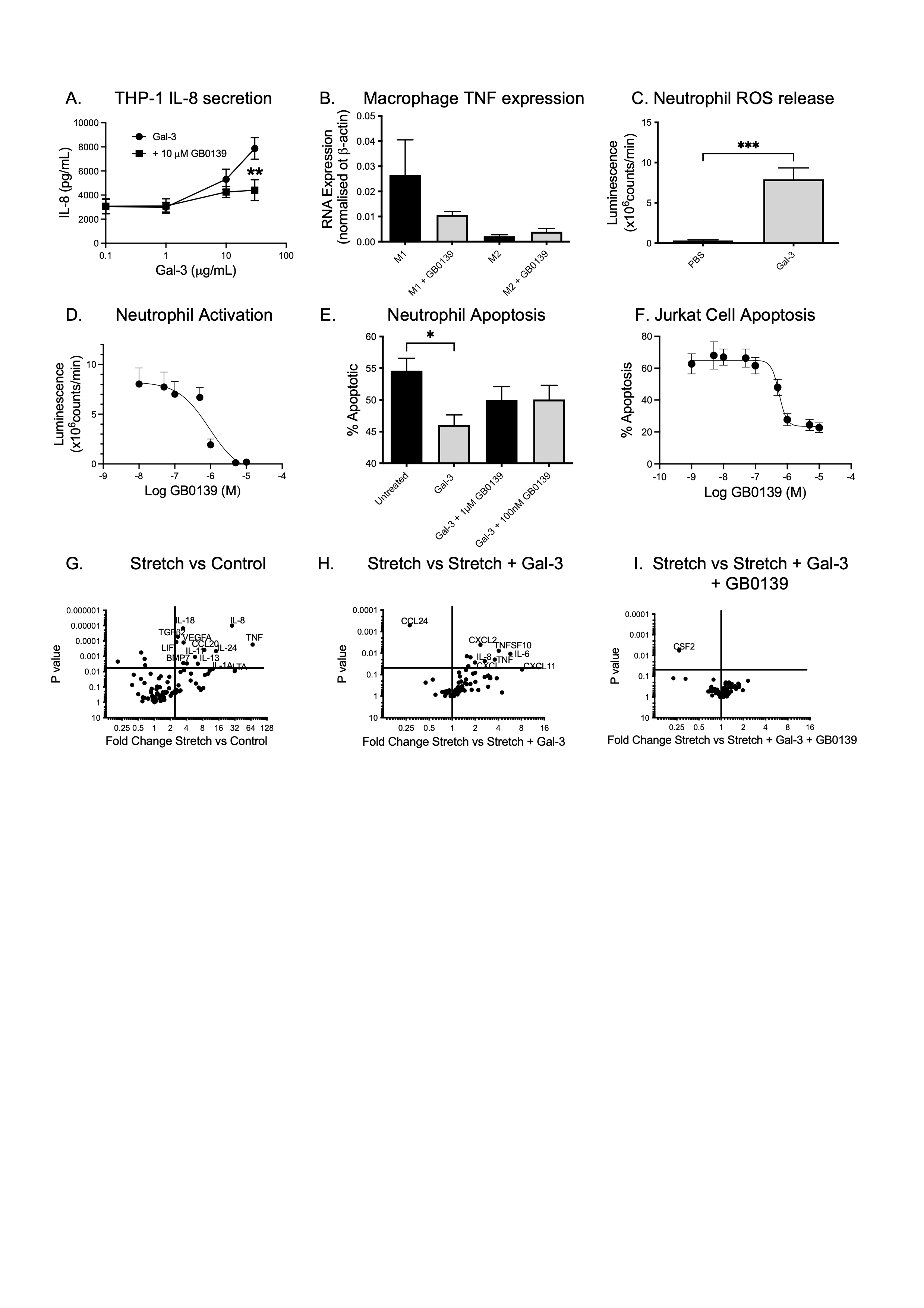

Supplement: Supplementary file 7 [file Presentation6.zip › figure 6.tiff]
