# Supplementary material for: Galectin-3 inhibitor GB0139 protects against acute lung injury by inhibiting neutrophil recruitment and activation
Source: Front Pharmacol. 2022 Aug 8;13:949264. doi: 10.3389/fphar.2022.949264 (PMC9393216; doi:10.3389/fphar.2022.949264)
Supplement: Supplementary file 5 [file DataSheet1.pdf]

Online Data Supplement

Galectin-3 Inhibitor GB0139 Protects Against Acute Lung Injury by Inhibiting Neutrophil Recruitment and Activation

Duncan C. Humphries, Ross Mills, Cecilia Boz, Brian J. McHugh, Nikhil Hirani, Adriano G. Rossi, Anders Pedersen, Hans T. Schambye, Robert J. Slack, Hakon Leffler, Ulf J. Nilsson, Wei Wang, Tariq Sethi, Alison C. Mackinnon

Intra-tracheal pharmacokinetics in the mouse: Pharmacokinetics (PK) by the *intra-tracheal* (*i.t.)* route was investigated in female C57Bl/6J mice (n=81, body weight 19-20 g) housed in standard holding cages and maintained in a controlled environment with free access to food and water. GB0139 was dosed via the *i.t.* route (50 µL/mouse) at 0.5 mg/kg and 1 mg/kg (equivalent to 10 and 20 µg total lung dose). Bronchoalveolar lavage fluid (BALF) was collected 2, 4, 8, 12, 24 and 48 hours post-dosing by aspirating 0.5 mL PBS slowly into the trachea using a flexible butterfly catheter via a 3-way tap before the fluid was slowly withdrawn. This was repeated 3 times and BALF kept on ice prior to centrifugation (2,000 x g for 5 mins at 4 °C) and the cell pellet and BALF separated. The cell pellet was snap frozen and stored at -70 °C until day of analysis. Blood samples were taken at 15 mins, 30 mins, 1, 2, 4, 8, 12, 24 and 48 hours post-dosing via the facial vein into K2-EDTA tubes and put on ice prior to centrifugation (2,000 x g for 5 mins at 4 °C) with plasma recovered, snap frozen and stored at -70 °C until day of analysis.

Plasma analysis: Plasma samples from *PK* studies were thawed and mixed with acetonitrile (1:8) containing internal standard and centrifuged at 1500 x *g* for 10 minutes. Mouse

bronchoalveolar lavage (BAL) cells were thawed and reconstituted in water (1 x 10^6 cells/mL) then mixed with acetonitrile (1:8) containing internal standard and centrifuged at 1500 x g for 10 minutes. The concentration of GB0139 in blood and BAL cells was then determined by liquid chromatography-tandem mass spectrometry (LC-MS/MS).

Induction of Acute Lung Injury: To induce pulmonary inflammation/fibrosis, mice received 10 µg lipopolysaccharide (LPS, serotype 0127:B8, L4516, Sigma-Aldrich) from *E. coli* or 33 µg bleomycin (BI3543, Apollo Scientific, UK) via an *intra-tracheal* (*i.t.*) route. Briefly, mice were anaesthetized with either Ketamine (75 mg/kg, Vetalar, Boehringer) & Medetomidine (1 mg/kg, Domitor, Pfizer) via *intra-peritoneal* injection or inhaled Isoflurane (Zoetis, UK) prior to the insertion of a blunted metal 23-gauge intra-tracheal needle. LPS/Bleomycin ± GB0139 (in 50 µL 0.9% NaCl), was inserted into the needle via a pipette, and delivered into the lungs with a 2 x 100 µL bolus of air (using a 1 mL syringe). As a control, mice were given 50 µL PBS (*i.t.*). The needle was then removed, and the mouse kept upright to ensure entry into the lungs. To reverse anaesthesia, Atipamezole (1 mg/kg, Antisedan, Pfizer) was injected *sub-cutaneously* 20 minutes after induction of anaesthesia, and animals left to recover at 29 °C overnight prior to retrieval.

Bronchoalveolar Lavage: BALF was obtained by exposing the trachea and inserting a plastic-coated 25-gauge needle which was secured in place with elasticated thread. 3 boluses of 0.8 mL PBS were then instilled and retrieved prior to being stored on ice. The first bolus was kept separate from the subsequent two. Lavages were weighed to establish total volume, before being centrifuged at 350 x g for 5 minutes. Supernatants were removed and stored at -80 °C prior to cytokine/protein analysis. Cell pellets were then combined in 1 mL PBS prior to

performing a differential cell count on cytocentrifuged preparations stained with Quick-Diff kit (102164, Reagena, West Sussex, UK).

Histology and Immunohistochemistry Preparation: Following exsanguination and retrieval of BALF, lungs were perfused with 10 mL PBS through the right ventricle. Lungs were quickly dissected free and, after tying off the left bronchiole, the right lung was inflated with formalin (HT501128, Sigma-Aldrich) via the trachea and stored for 24 hours before being transferred to 70% ethanol. Lungs were then paraffin-wax embedded prior to being sectioned and stained with haematoxylin and eosin or Masson's trichrome. Total inflammation score and fibrosis score was assessed according to published protocols (Murao et al., 2003; Hübner et al., 2008).

Flow Cytometric Analysis of Lung Digests: Following exsanguination and retrieval of BALF, lungs were perfused with 10 mL PBS through the right ventricle. After tying off the left bronchiole, the left lung was dissected free and placed in a DNase (DNase 1, 1 mg/mL, DN-25, Sigma-Aldrich Company Ltd) and Collagenase (Collagenase D, 10 mg/mL, 11088866001, Roche) mix before being disrupted with scissors and incubated at 37 ^{o}C for 1 hour. Cells were further released from tissue by vigorous pipetting using a 1 mL syringe and centrifuged at 300 x g for 15 minutes. The pellet was resuspended in 3 mL cold ACK buffer (Ammonium-Chloride-Potassium, A10492-01, Invitrogen, Carlsbad, California, USA) for 5 minutes on ice to lyse red cells before adding 2 mL PBS and centrifuged at 300 x g for 5 minutes. Following another wash, cells were resuspended in PBS and strained using a 40 µm cell strainer (352340, BD Biosciences). Fc block^{TM} was added 1:100 to the lung suspension for 10 minutes at 4 ^{o}C prior to another wash in PBS at 300 x g for 5 minutes. Cells were resuspended in antibody mix in PBS and stained on ice for 30 minutes before the addition of lysis fixation solution (349202, BD Europe). Samples were centrifuged at 350 x g for 5 minutes and resuspended in PBS prior

to analysis. The cytometer was set to collect 10,000 events (LSR Fortessa, BD Biosciences). Data analysis was performed using FlowJo software, version 7.2.4 (Tree Star Inc., USA).

The following flow cytometry antibody panels were used;

- *Macrophage and dendritic cell subsets in the mouse lung* (Misharin et al., 2013) - eFluoro-450 anti-mouse CD11b, 48011282, Ebiosciences; PE-Cy7 anti-mouse CD11c, 558079, BD Biosciences; APC anti-mouse CD24, 17024282, Ebiosciences; BV650 anti-mouse CD45, 11045182, Ebiosciences; PE anti-mouse CD64, 139304, Biolegend; APC-Cy7 anti-mouse LY-6C, 560596, BD Biosciences; Alexa-Fluor 700 anti-mouse LY-6G, 561236, BD Biosciences; PerCP-Cy5.5 anti-mouse MHC II, 562363 BD Biosciences; Live/Dead fixable aqua, L34965, ThermoFisher Scientific.
- *M1/M2 panel* - eFluoro-450 anti-mouse CD11b, 48011282, Ebiosciences; AF700 anti-mouse CD11c antibody, 117320, Biolegend; PE anti-mouse CD80, 104708, Biolegend; PE-Cy7 anti-mouse CD206, 141719, Biolegend; APC anti-mouse Siglec-F (CD170), 155507, Biolegend.
- *Lymphocyte panel*- PE-Cy7 anti-mouse CD3, 100220, Biolegend; PE anti-mouse CD4, 100408, Biolegend; Alexa Fluor 700 anti-mouse CD8, 100730, Biolegend; Pacific Blue anti-mouse B220, 103227, Biolegend.
- *Neutrophil activation panel*- Alexa Fluor 700 anti-mouse LY-6G, 561236, BD Biosciences; eFluoro-450 anti-mouse CD11b, 48011282, Ebiosciences; PE anti-mouse CD62-L. 104408, Biolegend.

Determination of lung collagen by sircol assay: The left lobe was minced in 5 mL of 3 mg/mL pepsin in 0.5 M acetic acid and incubated with shaking at 4 °C for 24 hours. Cleared lung extract (0.2 mL) was incubated with 0.8 mL sircol reagent for 1 hour at room temperature and

precipitated collagen centrifuged at 10,000 x g for 5 min at 4 °C. Pellets were solubilized in 1 mL 1 M NaOH and absorbance measured at 570 nm alongside collagen standards.

Macrophage RNA Analysis: Human monocyte-derived macrophages were cultured in the presence of GM-CSF (215-GM, R&D Systems, Minneapolis, MN) or M-CSF (216-MC, R&D Systems) for 6 days and then further activated with IFN-γ (285-IF, R&D) and LPS (L4516, Sigma-Aldrich) or IL-4 (404-ML, R&D) ± 10 µM GB0139 for 48 hours. Total RNA from cultured cells was prepared using RNeasy kits (Qiagen, Hilden, Germany) and reverse transcribed into cDNA using Quantitect RT kits (Qiagen). cDNA was analysed using a SYBR green-based quantitative fluorescence method (Invitrogen) and Kiqstart primers (Sigma Aldrich).

PCR: RNA from A549 cells was analysed using the human chemokines & cytokines RT2 Profiler PCR Array (SABiosciences PAHS-150Z, Germany) as per manufacturer's instructions (*ADIPOQ, BMP2, BMP4, BMP6, BMP7, C5, CCL1, CCL11, CCL13, CCL17, CCL18, CCL19, CCL2, CCL20, CCL21, CCL22, CCL24, CCL3, CCL5, CCL7, CCL8, CD40LG, CNTF, CSF1, CSF2, CSF3, CX3CL1, CXCL1, CXCL10, CXCL11, CXCL12, CXCL13, CXCL16, CXCL2, CXCL5, CXCL9, FASLG, GPI, IFNA2, IFNG, IL10, IL11, IL12A, IL12B, IL13, IL15, IL16, IL17A, IL17F, IL18, IL1A, IL1B, IL1RN, IL2, IL21, IL22, IL23A, IL24, IL27, IL3, IL4, IL5, IL6, IL7, CXCL8, IL9, LIF, LTA, LTB, MIF, MSTN, NODAL, OSM, PPBP, SPP1, TGFB2, THPO, TNF, TNFRSF11B, TNFSF10, TNFSF11, TNFSF13B, VEGFA XCL1*). Data analysis was carried out using an integrated web-based software package (SABiosciences) based on the $2^{-\Delta\Delta CT}$ method with normalization of the raw data to GapDH and is expressed as fold regulation.

Supplementary figures

Supplementary Figure 1

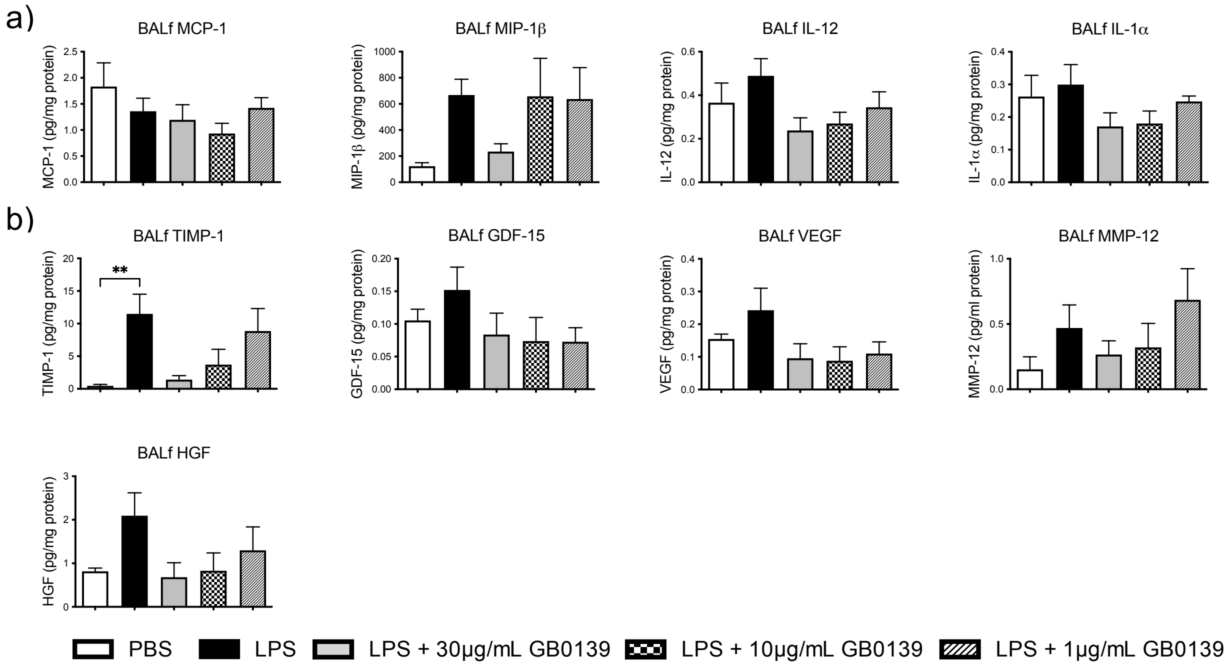

Supplementary Figure 1: *BALf cytokine quantification*. Cytokine levels were normalised against total BALf protein. a) BALf pro-inflammatory cytokine profiles following 10 µg LPS + 30 µg GB0139. BALf was collected 24 hours after LPS administration and cytokines quantified using the luminex array. b) BALf fibrosis-associated cytokine profiles. Data represented as mean ± SEM. Analysed via 1-way ANOVA (n=6, *p<0.05). ANOVA = analysis of variance; BALf = broncho-alveolar lavage fluid; GDF = growth/differentation factor; HGF = hepatocyte growth factor; IL = interleukin; LPS = lipopolysaccharide; MCP = monocyte chemoattractant protein; MIP-1β = macrophage inflammatory protein-1-beta; MMP = matrix metallopeptidase; SEM = standard error of the mean; TIMP = tissue inhibitor of metalloproteinase; VEGF = vascular endothelial growth factor.

Supplementary Figure 2

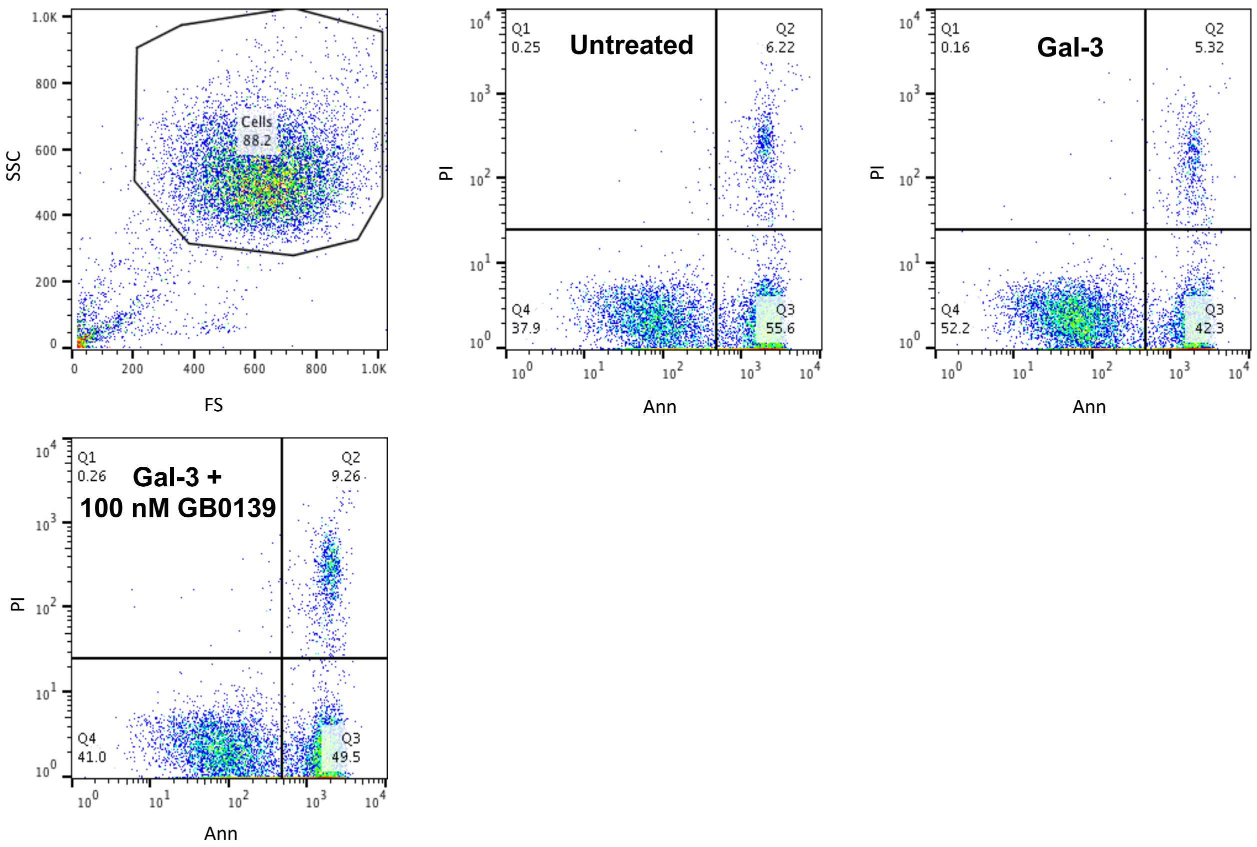

Supplementary Figure 2: *Neutrophil apoptosis following Gal-3 incubation*. After excluding cellular debris, rates of cell apoptosis/necrosis were calculated based on their Annexin V/PI expression. The same technique was applied to Jurkat cells.

Supplementary Figure 3

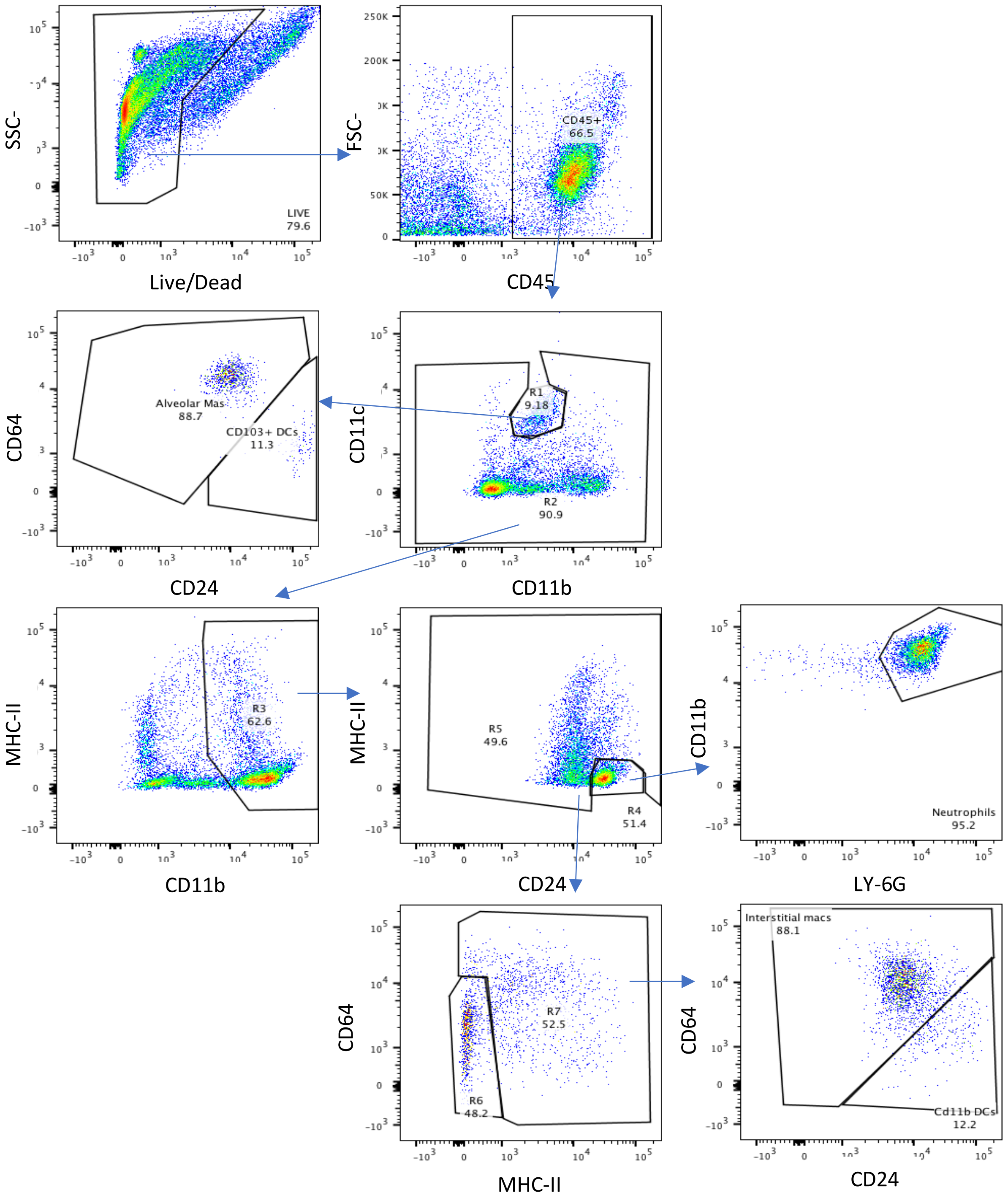

Supplementary Figure 3: *Flow cytometric gating strategy to identify immune cell subsets within the lung.* After gating for single, live cells, leukocytes were selected based on CD45 positive expression. 2 populations were gated based on their CD11b vs CD11c expression (R1 + R2) - cells from gate R1 were differentiated into alveolar macrophages and $CD103^+$ dendritic cells based on their CD24 vs CD64 expression. Cells from gate R2 were gated based on their CD11b vs MHC-II expression to form gate R3. Granulocytes were selected (R4) against remaining cells (R5) based on their CD24 and MHC-II expression. Cells from R4 were gated on their LY-6G vs CD11b expression to identify neutrophils. Cells from gate R5 were gated based on their MHC-II vs CD64 expression and further divided based on their CD24 and CD64 expression to identify interstitial macrophages and $CD11b^+$ dendritic cells.

Supplementary Figure 4

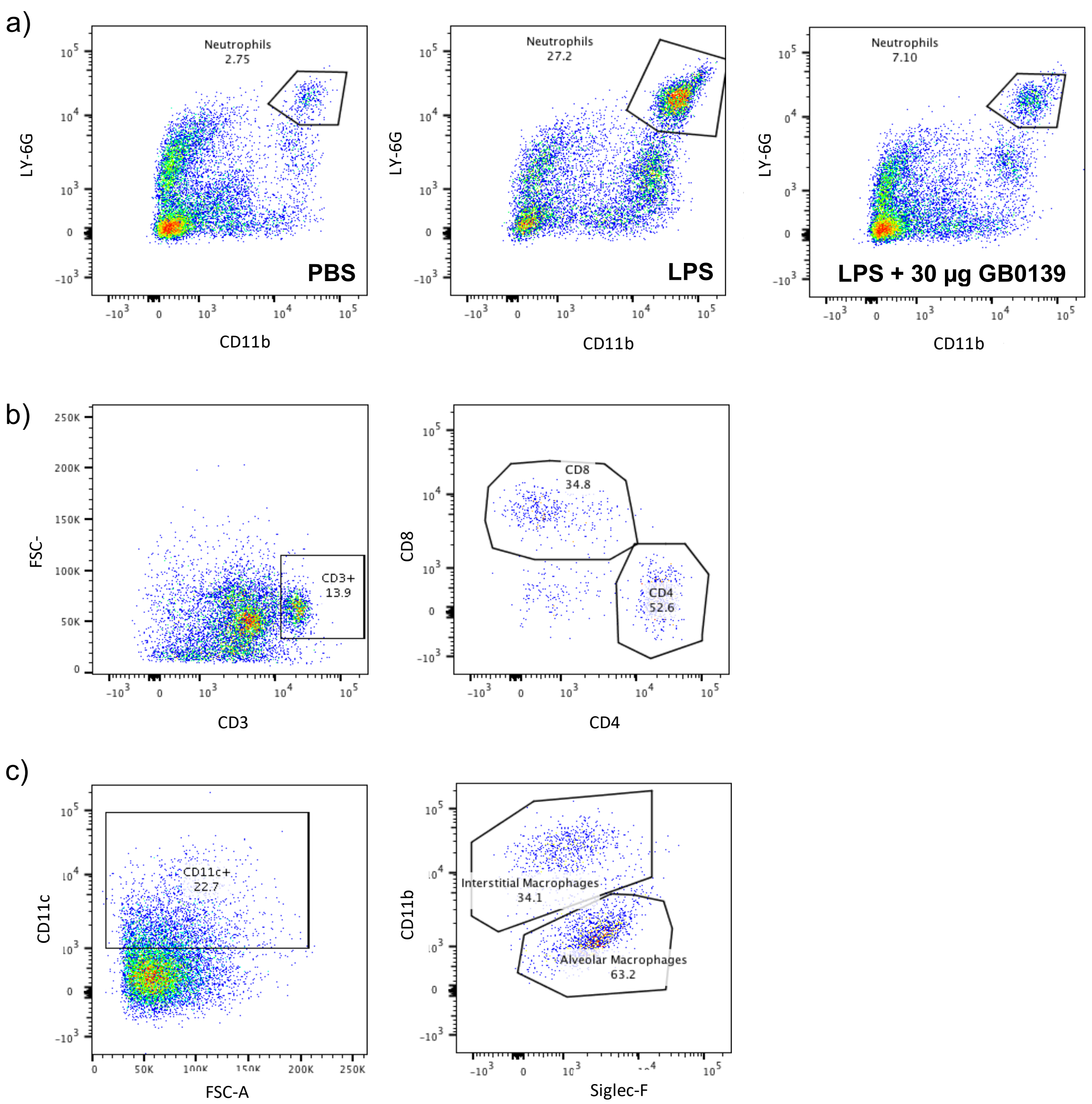

Supplementary Figure 4: *Flow cytometric gating strategies for identifying and phenotyping neutrophils, lymphocytes and macrophages.* a) Neutrophil gating strategy. After gating for single cells, neutrophils ($CD11b^{+}$, $LY\text{-}6G^{+}$) were selected.b) CD8 T-cell gating strategy. After gating for single cells, T lymphocytes were selected ($CD3^{+}$) and differentiated into cytotoxic T-cells ($CD3^{+}$, $CD8^{+}$) or helper T-cells ($CD3^{+}$, $CD4^{+}$). c) Alveolar/Interstitial Macrophage M1/M2 gating strategy. Alveolar macrophages ($CD11c^{hi}$, $CD11b^{low}$ Siglec F^{hi}) and interstitial macrophages ($CD11c^{int\text{-}hi}$, $CD11b^{hi}$, Siglec F^{low}) were selected and their average CD80/CD206 expression quantified.

Supplementary Figure 5

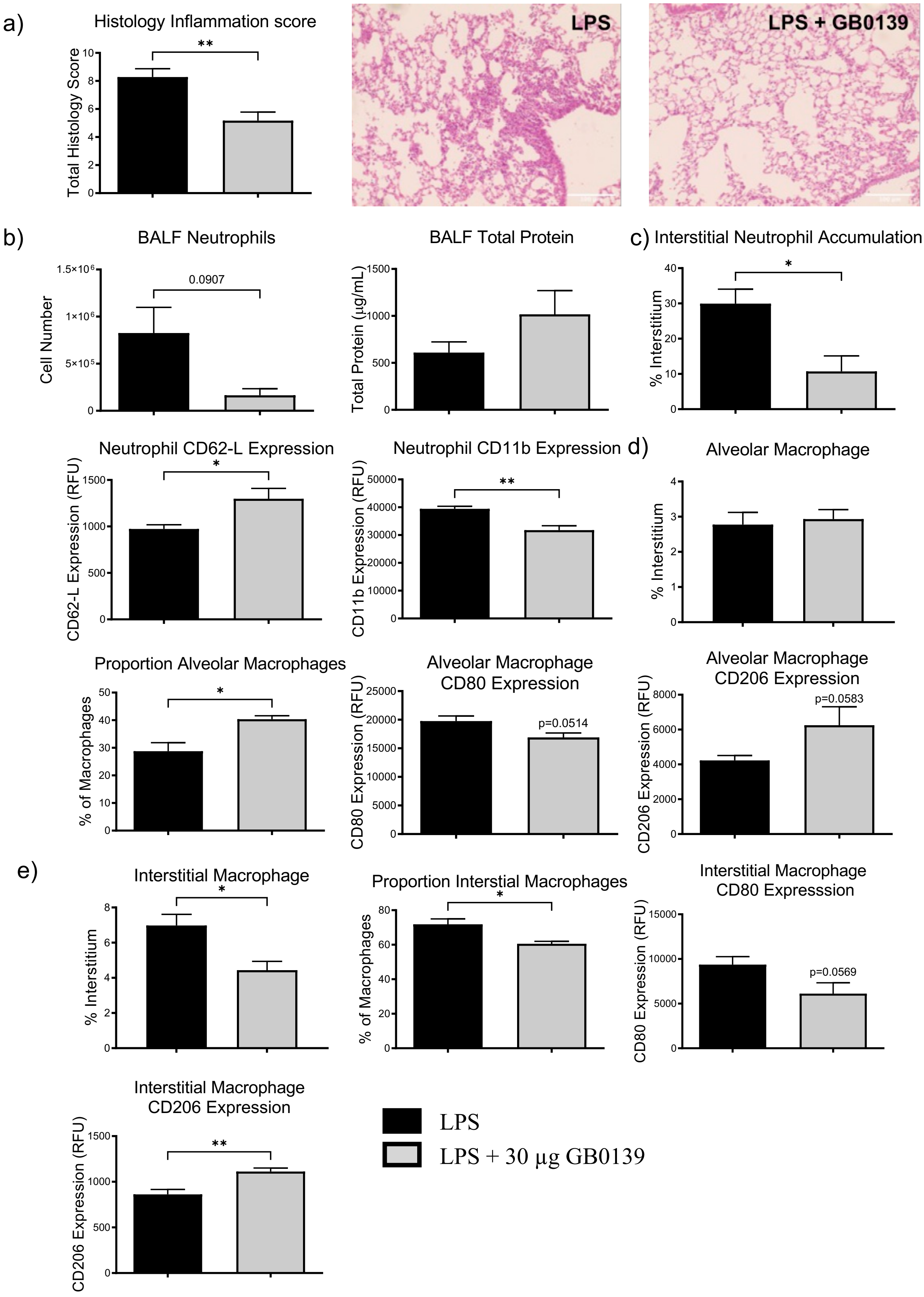

Supplementary Figure 5: *LPS + GB0139 (48 hour timepoint*). 10 µg LPS was administered alongside 30 µg GB0139 *intra-tracheally*. 24 hours later, a second dose of 30 µg GB0139 was delivered prior to retrieval at 48 hours. a) Histology inflammation score plus example images. b) Alveolar space characterisation. Alveolar neutrophils and total protein levels were quantified within the BALF. c) Interstitial neutrophil characterization. Neutrophil numbers and activation parameters were quantified via flow cytometry following tissue digest. d) Alveolar macrophage characterization. Alveolar macrophages (identified as $CD11c^{hi}$, $CD11b^{low}$ Siglec F^{hi}) and phenotype (M1/M2) were quantified via flow cytometry following tissue digest. e) Interstitial macrophage characterization. Interstitial macrophages (identified as $CD11c^{int\text{-}hi}$, $CD11b^{hi}$, Siglec F^{low}) and phenotype (M1/M2) were quantified via flow cytometry following tissue digest. Data represented as mean ± SEM. Analysed via students t-test (n=4-6, *$p<0.05$, **$p<0.01$). Images taken at x200.

Supplementary References:

Hübner, R. H., Gitter, W., el Mokhtari, N. E., Mathiak, M., Both, M., Bolte, H., et al. (2008). Standardized quantification of pulmonary fibrosis in histological samples. *Biotechniques* 44, 507–517. doi: 10.2144/000112729.

Misharin, A. v, Morales-Nebreda, L., Mutlu, G. M., Budinger, G. R. S., and Perlman, H. (2013). Flow Cytometric Analysis of the Macrophages and Dendritic Cell Subsets in the Mouse Lung. *Am J Respir Cell Mol Biol* 49, 503–510. doi: 10.1165/rcmb.2013-0086MA.

Murao, Y., Loomis, W., Wolf, P., Hoyt, D. B., and Junger, W. G. (2003). Effect of dose of hypertonic saline on its potential to prevent lung tissue damage in a mouse model of hemorrhagic shock. *Shock* 20, 29–34. doi: 10.1097/01.shk.0000071060.78689.f1.
